# Premedication before laryngoscopy in neonates: Evidence-based statement from the French society of neonatology (SFN)

**DOI:** 10.3389/fped.2022.1075184

**Published:** 2023-01-04

**Authors:** Xavier Durrmeyer, Elizabeth Walter-Nicolet, Clément Chollat, Jean-Louis Chabernaud, Juliette Barois, Anne-Cécile Chary Tardy, Daniel Berenguer, Antoine Bedu, Noura Zayat, Jean-Michel Roué, Anne Beissel, Claire Bellanger, Aurélie Desenfants, Riadh Boukhris, Anne Loose, Clarisse Massudom Tagny, Marie Chevallier, Christophe Milesi, Manon Tauzin

**Affiliations:** ^1^ Neonatal Intensive Care Unit, Centre Hospitalier Intercommunal de Créteil, Créteil, France; ^2^ Université Paris Est Créteil, Faculté de Santé de Créteil, IMRB, GRC CARMAS, Créteil, France; ^3^ Neonatal Medicine and Intensive Care Unit, Saint Joseph Hospital, Paris, France; ^4^ University of Paris-Cité, CRESS, Obstetrical Perinatal and Pediatric Epidemiology Research Team, EPOPé, INSERM, INRAE, Paris, France; ^5^ Department of Neonatology, Hôpital Armand Trousseau, APHP, Sorbonne Université, Paris, France; ^6^ Division of Neonatal and Pediatric Critical Care Transportation, Hôpital Antoine Beclere, AP-HP, Paris - Saclay University Hospital, Clamart, France; ^7^ Department of Neonatology and Neonatal Intensive Care, CH de Valenciennes, Valenciennes, France; ^8^ Department of Neonatology and Neonatal Intensive Care, Centre Hospitalier Universitaire de Dijon, Dijon, France; ^9^ Department of Pediatric Anesthesia and Pediatric Transport (SMUR Pédiatrique), Hôpital des Enfants, CHU de Bordeaux, Bordeaux, France; ^10^ Department of Neonatal Pediatrics and Intensive Care, Limoges University Hospital, Limoges, France; ^11^ Department of Neonatal Intensive Care and Pediatric Transport, CHU de Nantes, Nantes, France; ^12^ Department of Pediatric and Neonatal Critical Care, Brest University Hospital, Brest, France; ^13^ Neonatal Intensive Care Unit, Hôpital Femme Mère Enfant, Hospices Civils de Lyon, Bron, France; ^14^ Department of Neonatology and Neonatal Intensive Care, AP-HP, Hôpital Necker-Enfants Malades, Paris, France; ^15^ Department of Neonatology, CHU Nimes, Université Montpellier, Nimes, France; ^16^ Department of Neonatology, Pôle Femme-Mère-Nouveau-Né, Hôpital Jeanne de Flandre, Centre Hospitalier Universitaire de Lille, Lille, France; ^17^ Department of Neonatology, CHRU de Tours, Hôpital Bretonneau, Tours, France; ^18^ Department of Neonatology and Neonatal Intensive Care, Grand Hôpital de L’Est Francilien, Meaux, France; ^19^ Department of Neonatal Intensive Care Unit, CHU Grenoble, Grenoble, France; ^20^ TIMC-IMAG Research Department, Grenoble Alps University, Grenoble, France; ^21^ Department of Neonatal Medicine and Pediatric Intensive Care, Montpellier University Hospital, Université de Montpellier, Montpellier, France

**Keywords:** neonate, analgeisa, sedation, anesthesia, intubation (intratracheal), less invasive surfactant administration (LISA), laryngeal mask, atropine

## Abstract

**Context:**

Laryngoscopy is frequently required in neonatal intensive care. Awake laryngoscopy has deleterious effects but practice remains heterogeneous regarding premedication use. The goal of this statement was to provide evidence-based good practice guidance for clinicians regarding premedication before tracheal intubation, less invasive surfactant administration (LISA) and laryngeal mask insertion in neonates.

**Methods:**

A group of experts brought together by the French Society of Neonatology (SFN) addressed 4 fields related to premedication before upper airway access in neonates: (1) tracheal intubation; (2) less invasive surfactant administration; (3) laryngeal mask insertion; (4) use of atropine for the 3 previous procedures. Evidence was gathered and assessed on predefined questions related to these fields. Consensual statements were issued using the GRADE methodology.

**Results:**

Among the 15 formalized good practice statements, 2 were strong recommendations to do (Grade 1+) or not to do (Grade 1−), and 4 were discretionary recommendations to do (Grade 2+). For 9 good practice statements, the GRADE method could not be applied, resulting in an expert opinion. For tracheal intubation premedication was considered mandatory except for life-threatening situations (Grade 1+). Recommended premedications were a combination of opioid + muscle blocker (Grade 2+) or propofol in the absence of hemodynamic compromise or hypotension (Grade 2+) while the use of a sole opioid was discouraged (Grade 1−). Statements regarding other molecules before tracheal intubation were expert opinions. For LISA premedication was recommended (Grade 2+) with the use of propofol (Grade 2+). Statements regarding other molecules before LISA were expert opinions. For laryngeal mask insertion and atropine use, no specific data was found and expert opinions were provided.

**Conclusion:**

This statement should help clinical decision regarding premedication before neonatal upper airway access and favor standardization of practices.

## 1. Introduction

Upper airway access is an essential aspect of neonatal critical care. Laryngoscopy is necessary during tracheal intubation and during administration of surfactant by a so-called “less invasive” method (LISA for less invasive surfactant administration or MIST for minimally invasive surfactant treatment). The insertion of a laryngeal mask also represents an invasive access to upper airways. The physiological effects of awake laryngoscopy in neonates have been known since the 1980s and involve sudden changes in heart rate, blood pressure, oxygen saturation and intracranial pressure (1–3). These different phenomena raise fears that awake intubation may play a role in the occurrence of intraventricular hemorrhage in premature neonates (4, 5). Finally, the painful, stressful and uncomfortable nature of awake laryngoscopy is consensual among neonatal caregivers (6, 7). In adults, nasal insertion of an endotracheal tube (ETT) and the experience of artificial ventilation without sedation are sources of intense pain and stress (8–10). Several academic societies have therefore recommended the use of sedation, and/or analgesia, and/or anesthesia before neonatal intubation, except in an immediate life-threatening situation (11–13).

Nevertheless, practices remain very heterogeneous and awake intubation remains common in many neonatal departments (14–16), especially in the delivery room (17). Regarding the LISA or MIST methods, premedication practices are also very heterogeneous from one country or unit to another (18–22). However, this technique requires the performance of a laryngoscopy, whose harmful effects have been mentioned previously. Finally, the use of the laryngeal mask is now becoming a technique that can be used in newborns, even if its indications remain to be specified (23). The discomfort and pain that can be caused by the insertion of this device also justify to discuss premedication.

The objective of this work is to provide updated good practice advice for premedication before neonatal laryngoscopy based on evidence from medical publications.

## 2. Context

### 2.1. Current barriers to premedication before laryngoscopy

The most commonly given reason to justify the absence of premedication before access to the upper airways in neonates is the fear that the molecules used might cause immediate and long-term side effects (24). The respiratory depressant effects of morphine (25) and hypotensive effects of sedatives (26, 27) are one of the obstacles to their use. These immediate effects, added to the possible deleterious effects specific to these drugs on the developing brain (neuro-apoptosis, neurotoxicity) can also explain the reluctance to use them (4, 28). However, these arguments must be weighed against the immediate and long-term harmful effects of pain and stress associated with awake intubation or laryngoscopy (4, 29–32).

### 2.2. Safety and environmental/organizational conditions

Beyond the different molecules discussed in this text, it should be remembered that the management of access to the upper airways and the administration of sedative drugs, powerful analgesics and/or anesthetics should only be done under optimal safety conditions for the neonate. The immediate vital emergency situation therefore does not fall within the scope of this statement.

Regarding the environment, everything must be done to ensure maximum safety conditions, anticipating possible difficulties or complications related to the procedure and the patient. The following statements therefore only apply to an environment including:

- continuous monitoring of vital signs: heart rate, respiratory rate, arterial blood pressure and pulse oximetry (SpO_2_);
- the presence of a sufficient number of competent personnel in the care of the neonate and his or her pathologies;
- the availability and proper functioning of all necessary equipment to access upper airways and provide effective assisted ventilation.

These conditions can be met, depending on local organization, in variable locations: delivery room, intensive care unit, and mobile neonatal transport teams. For this reason, the good practice statement presented here does not distinguish between different procedures depending on the place of care.

**Figure 1:**
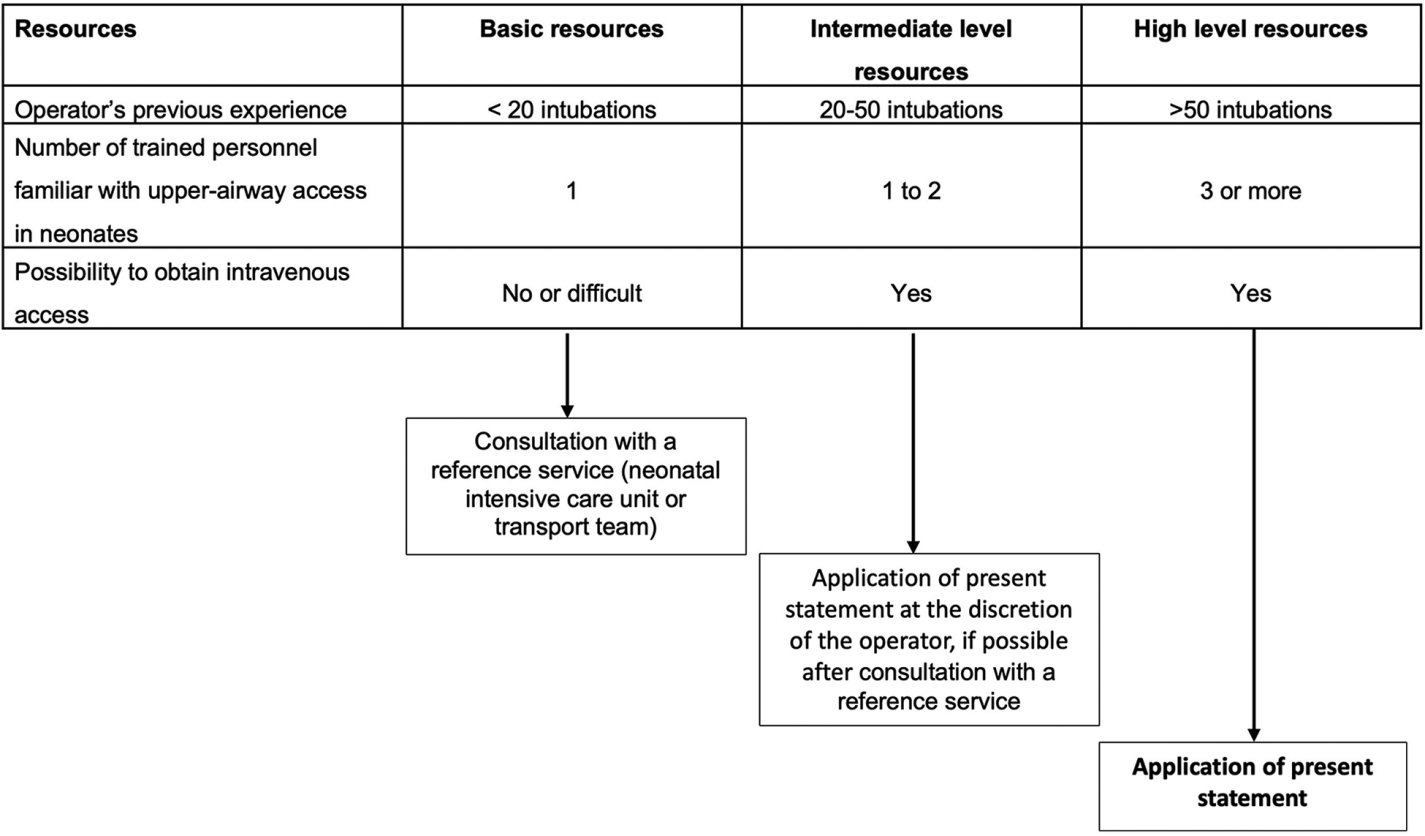
Conditions of statement application based on available resources.

The different resources available are presented in Figure 1. The present statement applies to an environment with high level resources. In other cases, the modalities of access to the upper airways are left to the discretion of the caregivers, in consultation with a reference service (intensive care unit or transport team) if possible.

## 3. Assessment of evidence-based good practice options and implications

### 3.1. Methods

This evidence-based good practice statement results from the work of a group of experts brought together by the French Society of Neonatology (SFN). The group's agenda was set in advance. As a first step, the working group defined the issues to be discussed with the coordinators. The group then appointed the experts in charge of each of them. The questions were formulated in a PICO (Patients Intervention Comparison Outcome) format after an initial meeting of the expert group. The terms and databases used for the literature search are provided in the Supplement. No limitation was applied for the publication date. Only randomized, controlled trials were selected, analyzed and summarized in GRADE evidence profiles tables. Retrospective and prospective observational studies were used to provide additional information but were not reported in tables. Case reports or reviews were not considered to build this statement. The analysis of the literature and the formulation of statements on good practice were then carried out according to the GRADE (Grading of Recommendation Assessment, Development and Evaluation) methodology (33). Table 1 summarizes the classification used for the level of evidence (LoE) and for the strength of recommendations. A LoE was defined for each of the bibliographical references cited according to the study's design and methodology. This LoE could be reassessed taking into account the methodological quality of the study (high, moderate, low or very low). An overall LoE was determined for each judgment criterion taking into account the levels of proof of each of the bibliographical references, the consistency of the results between the different studies and the direct nature or not of the evidence. A high or moderate overall LoE made it possible to formulate a strong recommendation (“must do, not do…”, GRADE 1+ or 1−). An overall moderate, low or very low LoE led to the issuance of a discretionary recommendation (“probably should/should not be done…”, GRADE 2+ or 2−). When the literature was non-existent or insufficient, the question could be the subject of a statement in the form of an expert opinion (“the experts suggest…”). The proposals for statements on good practice were presented and discussed one by one. The aim was not necessarily to arrive at a single, convergent expert opinion on all the proposals, but to identify the points of agreement and the points of divergence or indecision. Each good practice statement was then evaluated by each of the experts and submitted to their individual ratings using a scale ranging from 1 (complete disagreement) to 9 (complete agreement). The collective rating was established according to a GRADE grid methodology. To validate a good practice statement on a criterion, at least 50% of the experts had to express an opinion that generally went in the same direction, while less than 20% of them expressed a contrary opinion. For a good practice statement to be strong, at least 70% of the participants had to have an opinion that was broadly in the same direction. In the absence of strong agreement, the statement on good practice was reformulated and, again, submitted for rating with the aim of reaching a consensus. Finally, only the opinions of experts who obtained strong agreement could be retained.

**Table 1:** Summary of level of evidence and strength of recommendation classification according to the GRADE methodology.

Level of evidence
Grade	Definition	Letter
High	We are very confident that the true effect lies close to that of the estimate of the effect.	A
Moderate	We are moderately confident in the effect estimate: The true effect is likely to be close to the estimate of the effect, but there is a possibility that it is substantially different	B
Low	Our confidence in the effect estimate is limited: The true effect may be substantially different from the estimate of the effect.	C
Very low	We have very little confidence in the effect estimate: The true effect is likely to be substantially different from the estimate of effect	D
Strength of recommendation
Grade	Definition	Number
Strong for an intervention	“Clinicians must…”	1+
Discretionary for an intervention	“Clinicians probably should…”	2+
Discretionary against an intervention	“Clinicians probably should not…”	2−
Strong against an intervention	“Clinicians must not…”	1−

### 3.2. Selected domains

Four domains were defined *a priori*:

- premedication before tracheal intubation in neonates;
- premedication before intra-tracheal surfactant instillation without intubation (LISA or MIST) in neonates;
- premedication before laryngeal mask insertion in neonates;
- administration of atropine before upper airways access in neonates.

## 4. Actionable recommendations

### 4.1. Synthesis of results

The work of the experts and the application of the GRADE method resulted in 15 statements of good practice. Among the 15 formalized good practice statements, 2 were strong recommendations (GRADE 1+ and GRADE 1−) and 4 were discretionary recommendations (GRADE 2+). For 9 good practice statements, the GRADE method could not be applied, resulting in an expert opinion. After 1 round of rating and amendments, strong agreement was obtained for all statements. Table 2 summarizes these statements for tracheal intubation and the LISA procedure.

**Table 2:** Summary of good practice statements for premedication prior to neonatal laryngoscopy for tracheal intubation or LISA.

Question	Statement	Overall LoE	Strength of recommendation
**Endotracheal intubation**			
Should premedication be performed in neonates prior to tracheal intubation compared to awake intubation outside life-threatening emergencies?	Premedication must be performed in neonates prior to tracheal intubation outside life-threatening emergencies	B	1+
Can the combination of an opioid with a muscle blocker be used as premedication prior to tracheal intubation in neonates?	The combination of an opioid with a muscle blocker should probably be considered as possible premedication prior to tracheal intubation in neonates. Caveat: the use of muscle blocker eliminates all spontaneous ventilation and requires effective mask ventilation.	B	2+
Can a sole opioid be used as premedication prior to tracheal intubation in neonates?	Morphine or intravenous (IV) remifentanil alone must not be considered as premedication prior to tracheal intubation in neonates.	B	1−
Can IV midazolam alone be used as premedication prior to tracheal intubation in neonates?	The experts suggest avoiding the use of IV midazolam alone prior to tracheal intubation in neonates. The experts suggest that IV midazolam in combination with a rapid-acting synthetic opioid should be considered as a possible premedication prior to tracheal intubation in neonates.	C	Expert opinion
Can IV propofol be used as premedication prior to tracheal intubation in neonates?	IV propofol should probably be considered as a possible premedication prior to tracheal intubation in neonates.	B	2+
Can IV ketamine be used as premedication prior to tracheal intubation in neonates?	The experts suggest to consider IV ketamine as a possible premedication prior to tracheal intubation in neonates.	D	Expert opinion
If there is no venous access, can intranasal midazolam or ketamine be used as premedication in neonates prior to tracheal intubation?	The experts recommend that every effort should be made to establish a venous access prior to tracheal intubation in neonates. In the absence of a venous access, the experts suggest considering the intranasal administration of ketamine or midazolam as possible premedications, without it being possible to establish a preference between these 2 molecules.	C	Expert opinion
**Premedication before less-invasive surfactant administration (LISA)**			
Should neonates receive premedication prior to LISA?	Premedication should probably be administered prior to LISA.	B	2+
Can IV opioids be used as a premedication prior to LISA in neonates?	The experts suggest to consider IV fentanyl as a possible premedication prior to LISA in neonates.	C	Expert opinion
Can IV propofol be used as a premedication prior to LISA in neonates?	IV propofol should probably be considered as a possible premedication prior to LISA in neonates.	B	2+
Can IV ketamine be used as a premedication prior to LISA in neonates?	The experts suggest to consider IV ketamine as a possible premedication prior to LISA in neonates.	D	Expert opinion

LISA, less invasive surfactant administration; LoE, level of evidence.

### 4.2. Premedication before tracheal intubation in neonates

#### 4.2.1. Question 1: Should premedication be performed in neonates prior to tracheal intubation compared to awake intubation outside life-threatening emergencies?

**Statement**: Premedication must be performed in neonates prior to tracheal intubation outside life-threatening emergencies (strong recommendation).

*Precautions related to gestational age*: Studies were performed in term and preterm infants.

*Precautions on hemodynamic status*: In the event of extreme bradycardia or cardiac arrest, the priority is the initiation of effective ventilation and intubation should not be delayed by any other consideration.

**Rationale (Supplementary Table S1)**: Seven randomized controlled studies compared the premedication to awake intubation or the use of atropine alone before intubation: thiopental (*n* = 14) vs. control group (*n* = 13) (34); midazolam (*n* = 7) vs. 2 control groups: atropine + placebo (*n* = 6) and placebo alone (*n* = 3) (35); atropine + morphine + suxamethonium (*n* = 10) vs. control group (*n* = 10) (36); morphine alone (*n* = 17) vs. control group (*n* = 17) (37); sevoflurane (*n* = 19) vs. control group (*n* = 14) (38); atropine + remifentanil (*n* = 20) vs. atropine alone (*n* = 20) (39); and atropine + midazolam (*n* = 40) vs. atropine + placebo (*n* = 40) (40). The lack of methodological data concerning the first study on midazolam alone led to its exclusion from this analysis (35). The methodology of the study comparing atropine + midazolam vs. atropine + placebo (40) was very weak (ambiguous randomization, insufficiently detailed methods) requiring cautious interpretation of its results. Two older randomized studies evaluated the use of a muscle-blocker without associated sedo-analgesia or anesthesia (1, 41), but this practice has been discouraged for several years (12). These 2 studies were therefore not taken into account.

*Number of attempts*: Five studies evaluated the number of attempts or the rate of first attempt failure (34, 36, 37, 39, 40) and it was significantly reduced by the use of premedication in 2 of them (36, 40).

*Duration of the procedure*: Five studies evaluated the duration of the procedure (36–40) and in 3 of them premedication significantly reduced the duration of the procedure (34, 36, 40).

*Hypoxia*: All 6 selected studies evaluated the frequency of desaturations or SpO_2_ values. No study found a significant decrease in saturation associated with premedication. The frequency of desaturations was decreased by premedication in one study (40).

*Bradycardia*: Two studies evaluated the frequency of bradycardia (37, 38). No study found an increase in the frequency of bradycardia and one found a reduction in bradycardia with the use of premedication (38).

*Hypotension*: Three studies assessed blood pressure or hypotension (34, 38, 39). One study found a decrease in mean arterial pressure in the premedicated group and an increase in mean arterial pressure in the awake intubation group (34).

*Pain and comfort*: Two studies (very low quality) evaluated the pain of newborns and found a significant decrease in the premature infant pain profile (PIPP) (42) and faceless acute neonatal pain (FANS) (43) scores in the premedicated group (39, 40).

*Intubation conditions*: Two studies evaluated the technical conditions of intubation and both found better conditions in the groups receiving premedication (38, 40).

**Summary (Moderate LoE)**: Although the analyzed studies included small numbers of patients and had questionable premedication regimens (see below), the practice of awake intubation in neonates potentially exposes to an increased risk of failure and/or prolongation of the duration of the procedure. In addition, premedication prior to intubation does not result in more desaturations or bradycardia. The effect on blood pressure is dependent on the molecule used and will be discussed for each evaluated regimen. Intubation conditions for the infant and the operator might be improved by premedication. Last but not least, current knowledge on the existence and deleterious nature of pain in neonates imposes the use of sedo-analgesia or anesthesia before intubation, except in the case of an immediate life-threatening emergency. However, the risk-benefit profile of the chosen drug(s) must be assessed carefully for each specific infant. The following statements will help clinicians to estimate this profile for each assessed regimen.

#### 4.2.2. Question 2: Can the combination of an opioid with a muscle blocker be used as premedication prior to tracheal intubation in neonates?

**Statement**: The combination of an opioid with a muscle blocker should probably be considered as possible premedication prior to tracheal intubation in neonates (discretionary recommendation). Caveat: the use of muscle blocker eliminates all spontaneous ventilation and requires effective mask ventilation.

*Precautions related to gestational age*: Studies were performed in term and preterm infants.

*Precautions on hemodynamic status*: No specific limitation.

**Rationale (Supplementary Table S2)**: Seven randomized controlled studies compared various combinations of opioids and muscle blockers to various other regimens: atropine + morphine + suxamethonium (*n* = 10) vs. no treatment (*n* = 10) (36); atropine + fentanyl + mivacurium (*n* = 21) vs. atropine + fentanyl (*n* = 20) (44); atropine + morphine + suxamethonium (*n* = 30) vs. propofol (*n* = 33) (45); atropine + fentanyl + suxamethonium (*n* = 15) vs. atropine + remifentanil (*n* = 15) (46); glycopyrrolate + thiopental + remifentanil + suxamethonium (*n* = 17) vs. atropine + morphine (*n* = 17) (47); atropine + fentanyl + rocuronium (*n* = 20) vs. atropine + fentanyl (*n* = 24) (48); atropine + sufentanil + atracurium (*n* = 82) vs. atropine + propofol (*n* = 89) (49).

*Number of attempts*: All 7 studies assessed the number of attempts or the rate of first attempt failure. In 2 studies, the success rate of the first one or two attempts was significantly higher with the opioid + muscle blocker combination (36, 44), although there was no statistically significant difference in the median or average number of attempts in 6 studies (36, 44–47, 49).

*Duration of the procedure*: Six studies evaluated the duration of the procedure (36, 44–47, 49). The opioid-muscle blocker combination was associated with a significantly shorter procedure time in 4 studies (36, 44, 47, 49) and a significantly longer time in one study (45).

*Hypoxia*: Six studies evaluated the frequency of desaturations or SpO_2_ values (36, 44–47, 49). One study found fewer desaturations <60% in the opioid + muscle blocker group, but no difference for other SpO_2_ thresholds (44). One study found significantly lower SpO_2_ values during intubation in the opioid + muscle blocker group (45). The other studies did not find a significant difference in the frequency of desaturations (36, 46, 47, 49).

*Bradycardia*: Six studies evaluated the frequency of bradycardia or change in heart rate (36, 44–46, 48, 49). None of them found a significant increase in bradycardia associated with the opioid + muscle blocker combination.

*Hypotension*: Five studies evaluated blood pressure or hypotension (44–47, 49). Two studies found a decrease in the frequency of hypotension or a higher mean arterial pressure following premedication with the opioid + muscle blocker combination (47, 49).

*Pain and comfort*: The paralyzing effect of muscle blockers makes any behavioral pain scale unusable.

*Intubation conditions*: Three studies assessed technical conditions during intubation and all 3 found better conditions in the groups that received a combination of opioid + muscle blocker (46, 47, 49). Cases of thoracic rigidity were described with the use of atropine + atracurium + sufentanil (49).

*Additional information*: Good intubation conditions were also reported in several observational studies (50–53). In an international multicenter observational study including more than 2,000 intubations, the use of muscle blockers was an independent variable for reduced risk of adverse events during intubation in neonatal intensive care units (54). In 2 observational studies, an increase in CO_2_ partial pressure was observed with the use of an opioid + muscle blocker combination (52, 55). Regarding neurodevelopmental outcome at age 2 (ASQ scores), an ancillary study of a randomized controlled trial showed no difference between atropine + propofol and atropine + sufentanil + atracurium (56).

**Summary (Moderate LoE)**: The combination of an opioid and a muscle blocker is the most studied premedication regimen for neonatal intubation. It facilitates the procedure and seems to reduce its duration. Data on tolerance are reassuring both in the short and long term, particularly for hemodynamics. Nevertheless, the paralyzing effect of muscle blockers makes any behavioral pain scale unusable, which is a limitation. Furthermore, the experts point out that the use of muscle blockers requires effective oxygenation and mask ventilation due to the suppression of all respiratory movements. Particular vigilance on these points is therefore necessary for operators who are not experienced in handling these molecules.

#### 4.2.3. Question 3: Can a sole opioid be used as premedication prior to tracheal intubation in neonates?

**Statement**: Morphine or intravenous (IV) remifentanil alone must not be considered as premedication prior to tracheal intubation in neonates (strong recommendation). There is no sufficient data on other opioids used alone in this indication.

*Precautions related to gestational age*: Studies were performed in term and preterm neonates.

*Precautions on hemodynamic status*: Not applicable.

**Rationale (Supplementary Table S3)**: Five randomized studies compared an opioid alone (morphine or remifentanil) with other strategies that may include opioids: morphine (*n* = 17) vs. placebo (*n* = 17) (37); atropine + remifentanil (*n* = 15) vs. atropine + fentanyl + suxamethonium (*n* = 15) (46); atropine + morphine (*n* = 17) vs. glycopyrrolate + thiopental + remifentanil + suxamethonium (*n* = 17) (47); atropine + remifentanil (*n* = 20) vs. atropine (*n* = 20) (39); atropine + remifentanil (*n* = 36) vs. atropine + morphine + midazolam (*n* = 35) (57).

*Number of attempts*: In these 5 studies, the number of attempts was not statistically different, whatever the opioid and the comparator (37, 39, 46, 47, 57).

*Duration of the procedure*: All 5 studies evaluated the duration of intubation (37, 39, 46, 47, 57). One study found a significant increase in the duration of intubation with the combination of atropine + morphine (47).

*Hypoxia*: All 5 studies evaluated the frequency of desaturations or SpO_2_ values (37, 39, 46, 47, 57). No clinically relevant differences were found.

*Bradycardia*: Three studies assessed the frequency of bradycardia or change in heart rate (37, 46, 57). None found an increase in bradycardia associated with the use of a sole opioid.

*Hypotension*: Three studies assessed blood pressure or hypotension (39, 46, 57). None found significant changes in blood pressure or in the frequency of hypotension associated with the use of a sole opioid.

*Pain and comfort*: For remifentanil, pain scores were decreased compared to placebo (39) and increased compared to morphine + midazolam (57).

*Intubation conditions*: Intubation conditions were evaluated in 3 studies (39, 46, 57). Intubation conditions assessed by the operator were significantly worse with remifentanil in one study (46). With remifentanil concerning episodes of chest rigidity were reported in two studies (39, 46) out of three.

*Additional information*: A randomized study compared morphine + midazolam (*n* = 10) vs. remifentanil + midazolam (*n* = 10) and found no significant difference on pain scores, but better intubation conditions according to the operator with remifentanil (58). A randomized study compared the combination of propofol + remifentanil (*n* = 10) vs. midazolam + remifentanil (*n* = 10) with good analgesic efficacy, good intubation conditions and good tolerance (59). Three observational studies were performed with remifentanil (60–62) and two of them (61, 62) reported concerning chest rigidities, responsible for the premature interruption of one of these studies (61).

**Summary (Moderate LoE)**: The pharmacokinetic of morphine does not support its use for premedication before tracheal intubation. Remifentanil seems to have an analgesic efficacy but its uncertain tolerance, notably because of the frequency of chest rigidity, advises against its use alone. Other synthetic opioids (fentanyl, sufentanil, alfentanil) have not been evaluated without associated muscle blocker.

#### 4.2.4. Question 4: Can IV midazolam alone be used as premedication prior to tracheal intubation in neonates?

**Statement**: The experts suggest avoiding the use of IV midazolam alone prior to tracheal intubation in neonates (Expert opinion). The experts suggest that IV midazolam in combination with a rapid-acting synthetic opioid should be considered as a possible premedication prior to tracheal intubation in neonates (Expert opinion).

*Precautions related to gestational age*: Two of the 3 selected randomized studies only included premature neonates born after 28 weeks of gestation (57, 59). Term infants were included in 1 study (57).

*Precautions on hemodynamic status*: Hemodynamic failure was a criterion for non-inclusion in only one of the 3 selected studies (59). Because of its hypotensive effect (63) and its 6 h half-life (64), midazolam does not appear to be appropriate in case of hemodynamic compromise.

**Rationale (Supplementary Table S4)**: Four randomized studies compared IV midazolam to other products: midazolam (*n* = 7) vs. 2 control groups: atropine + placebo (*n* = 6) and placebo alone (*n* = 3) (35); midazolam + remifentanil (*n* = 10) vs. propofol + remifentanil (*n* = 10) (59); atropine + midazolam + morphine (*n* = 35) vs. atropine + remifentanil (*n* = 36) (57); atropine + midazolam (*n* = 40) vs. atropine + placebo (*n* = 40) (40). The absence of methodological data concerning the first study on midazolam alone led to its exclusion from this analysis (35). Nevertheless, it should be noted that this study was stopped because of a high rate of cardiopulmonary resuscitation (29%) in the group allocated to midazolam (35). The methodology of the study comparing atropine + midazolam vs. atropine + placebo (40) was very weak (ambiguous randomization, insufficiently detailed methods), inviting cautious interpretation of its results.

*Number of attempts*: The 3 studies evaluated the number of attempts and one study (very low quality) found an increase in the success rate of the first attempt associated with the use of midazolam (40).

*Duration of intubation*: Two studies evaluated the duration of intubation (40, 57). One study (very low quality) found a reduction in intubation time associated with the use of midazolam (40).

*Hypoxia*: Two studies reported the frequency of desaturations or SpO_2_ values (40, 57). One study (very low quality) found a reduction in the frequency of desaturations associated with the use of midazolam (40). The other study (low quality) found a variable decrease for SpO_2_ in the post-intubation period associated with the use of the combination of atropine + midazolam + morphine (57).

*Bradycardia*: Two studies evaluated the occurrence of bradycardia (57, 59). No bradycardia was reported.

*Hypotension*: All 3 studies assessed blood pressure or hypotension (40, 57, 59). None found significant changes in blood pressure or the incidence of hypotension associated with the use of midazolam.

*Pain and comfort*: Two studies found a significant decrease in the pain scores using Acute Neonatal Pain (ANP) (65), PIPP (42) and FANS (43) scales in the midazolam group (40, 57) and one study found no difference with the compared product (propofol) (59) on the Neonatal Infant Pain Scale (NIPS) (66). However, since midazolam has no analgesic effect, it is possible that the decrease in behavioral pain scales was only due to a behavioral interference of this drug.

*Intubation conditions*: Intubation conditions were evaluated in all 3 studies (40, 57, 59). The conditions for the operator were only significantly improved with midazolam compared to placebo in one study (very low quality) (40).

*Additional information*: In 2 prospective observational studies, the combination of midazolam with a fast-acting opioids (sufentanil in one study, fentanyl in the other) was associated with good intubation conditions and proper sedation and analgesia (14, 67).

**Summary (low LoE)**: IV midazolam alone could be preferable to awake intubation. Nevertheless, only its combination with a fast-acting opioid guarantees its effectiveness in decreasing pain or discomfort scores. In addition, the purely sedative action of midazolam justifies its combination with an analgesic (68). The experts recommend that other modalities of premedication should be considered before using this molecule.

#### 4.2.5. Question 5: Can IV propofol be used as premedication prior to tracheal intubation in neonates?

**Statement**: IV propofol should probably be considered as a possible premedication prior to tracheal intubation in neonates (discretionary recommendation).

*Precautions related to gestational age*: Studies were performed in term and preterm infants. Dosage precaution according to weight (<1,000 g), probably start titration by increments of 0.5–1 mg/kg (69). Such doses require dilution of the product to ensure the accuracy of the administered dose.

*Precautions on hemodynamic status*: Contraindicated in cases of hemodynamic compromise or hypotension. Regular monitoring of blood pressure is mandatory.

**Rationale (Supplementary Table S5)**: Three randomized, controlled trials compared the use of propofol with other regimens: propofol (*n* = 33) vs. atropine + suxamethonium + morphine (*n* = 30) (45); propofol + remifentanil (*n* = 10) vs. midazolam + remifentanil (*n* = 10) (59) and atropine + propofol (*n* = 89) vs. atropine + atracurium + sufentanil (*n* = 82) (49).

*Number of attempts*: The 3 studies evaluated the number of attempts (45, 49, 59). None of them found a significant difference with the comparator concerning the number of attempts or the success rate of the first attempt.

*Duration of intubation*: Two studies evaluated the duration of intubation (45, 49). One study found a significant reduction (45) and the other a significant increase (49) in intubation time in the propofol group.

*Hypoxia*: Two studies reported the frequency of desaturations or SpO_2_ values (45, 49). One study found significantly higher SpO_2_ values during intubation in the propofol group (45).

*Bradycardia*: All 3 studies evaluated the occurrence of bradycardia (45, 49, 59). None found an increase in bradycardia associated with the use of propofol.

*Hypotension*: All 3 studies assessed blood pressure or arterial hypotension (45, 49, 59). Hypotension occurred significantly more frequently in the propofol group in one study (49).

*Pain and comfort*: In one study (59) there was no difference between the remifentanil + propofol and remifentanil + midazolam for the NIPS (66) and COMFORT (70) pain scores. The authors reported that both regimen were “capable of analgesia and sedation” although results were not detailed in the publication.

*Intubation conditions*: Intubation conditions were evaluated in 2 studies (49, 59). Intubation conditions with propofol were worse in one study (49) and unchanged in the other (59).

*Additional information*: In cohort studies, the frequency of hypotension was higher than in randomized trials and varied from 38% to 59% (27, 71, 72). Of note, studies of cerebral autoregulation assessed by Near Infra-Red Spectroscopy (NIRS), including an ancillary study of a randomized controlled trial (73) and a cohort study (74), did not find such frequent impairment of cerebral autoregulation, nor did they find a correlation between arterial hypotension and decreased oxygen delivery to the brain. An ancillary study of a randomized controlled trial showed no significant difference regarding neurodevelopmental outcome at 2 years (ASQ scores) between propofol and the combination of sufentanil + atracurium (56).

**Summary (moderate LoE)**: Propofol, although not an analgesic, seems to allow sufficiently deep sedation to ensure the comfort of the neonate during the procedure—as for other painful procedures in adults (75). In addition, its respiratory tolerance is better than that of the opioid + muscle blocker combination and the absence of paralysis allows individual titration to obtain the desired level of sedation. Its hypotensive effect contraindicates its use in case of proven or expected hemodynamic disorders. To date, no short- or medium-term neurotoxic effect has been reported.

#### 4.2.6. Question 6: Can IV ketamine be used as premedication prior to tracheal intubation in neonates?

**Statement**: The experts suggest to consider IV ketamine as a possible premedication prior to tracheal intubation in neonates (expert opinion).

*Precautions related to gestational age*: There are experimental data suggesting conflicting results on the neurotoxicity of ketamine (76, 77) with no clinical data on neurotoxicity.

*Precautions on hemodynamic status*: Lack of specific data, used in routine practice in older children and adults in case of hemodynamic compromise (76).

**Rationale**: No randomized study exists on ketamine in this setting. Only one prospective non-randomized study compared intubation in the delivery room with atropine + ketamine (*n* = 39) vs. awake intubation (*n* = 15) (78). The pain score was significantly lower with ketamine. No difference was observed in the duration of the procedure, number of attempts, changes in SpO_2_, and blood pressure or in-hospital morbidity. The prospective follow-up study of this cohort found no alert on neurodevelopment at 2 years (79).

**Summary (very low LoE)**: Ketamine has not been assessed in randomized, controlled studies in neonates, although it is regularly used in France as premedication, before neonatal intubation (17, 80). The experts recommend that other modalities of premedication should be considered before using this molecule.

#### 4.2.7. Question 7: If there is no venous access, can intranasal midazolam or ketamine be used as premedication prior to tracheal intubation in neonates?

**Statement**: Experts recommend that every effort should be made to establish a venous access prior to tracheal intubation in neonates. In the absence of a venous access, the experts suggest considering the intra-nasal administration of ketamine (nKTM) or midazolam (nMDZ) as possible premedications, without it being possible to establish a preference between these 2 molecules (expert opinion).

*Precautions related to gestational age*: The only randomized controlled study recruited infants born between 24 and 36 weeks of gestation.

*Precautions on hemodynamic status*: Same precautions as for the IV route, probably to be recommended for each of the products (midazolam and ketamine). The hemodynamic profile in the nKTM vs. nMDZ randomized trial was comparable in the 2 groups.

**Rationale (Supplementary Table S6)**: There is only one randomized controlled study comparing nKTM (*n* = 33) vs. nMDZ (*n* = 27) for tracheal intubation before exogenous surfactant instillation in the delivery room in preterm infants (81).

*Number of attempts*: The average number of attempts was not significantly different between the nMDZ and nKTM groups.

*Duration of intubation*: The duration of intubation was not significantly different between the nMDZ and nKTM groups.

*Hypoxia*: SpO_2_ nadir was not significantly different between nMDZ and nKTM groups.

*Bradycardia*: No bradycardia occurred in the nMDZ and nKTM groups.

*Hypotension*: Mean arterial pressure (MAP) nadirs and MAP kinetics were comparable between the nMDZ and nKTM groups.

*Pain and comfort*: Adequate sedation before intubation (TRACHEA score≤1 (14)) was significantly more frequent in the nMDZ group. Adequate comfort during intubation [FANS score <4 (43)] was comparable in both groups.

*Intubation conditions*: The combination of adequate pre-intubation sedation (TRACHEA score≤1 (14)) and adequate comfort during intubation [FANS score <4 (43)] was statistically more frequent in the nMDZ group.

*Additional information*: An observational study showed the feasibility of nMDZ (*n* = 27) before tracheal intubation in the delivery room in preterm infants born between 27 and 33 weeks of gestation with a satisfactory FANS score (82). A pain reaction was observed at the instillation of nMDZ in 30% of the children (acidity of the product).

**Summary (low LoE)**: nMDZ or nKTM are feasible in the absence of an injectable alternative. However, safety conditions are not optimal in the absence of a venous access and efforts should be focused on establishing a venous access before considering the intra-nasal route.

### 4.3. Premedication before less-invasive surfactant administration (LISA) in neonates

#### 4.3.1. Question 1: Should neonates receive premedication prior to LISA?

**Statement**: Premedication should probably be administered prior to LISA in neonates (discretionary recommendation).

*Precautions related to gestational age*: Published randomized trials included preterm neonates born at 26 weeks of gestation or above.

*Precautions on hemodynamic status*: No data are available on this point.

**Rationale (Supplementary Table S7)**: Two randomized studies have been published: propofol (*n* = 42) vs. no treatment (*n* = 36) (83) and fentanyl (*n* = 17) vs. no treatment (*n* = 17) (84).

*LISA failure*: Failure of the procedure was defined as intubation within 24 h (83) or 72 h (84) of the procedure. Intubation rates were not significantly increased by sedation-analgesia in these 2 studies (83, 84).

*Number of attempts*: The number of laryngoscopy attempts for LISA was not significantly modified by premedication use in these 2 studies (83, 84).

*Duration of the procedure*: Only one study evaluated the duration of the procedure and did not find any modification of this duration by premedication (83).

*Hypoxia*: The 2 studies evaluated the occurrence of desaturations. Only one study found a significant increase in the frequency of desaturations in the premedication group (83).

*Bradycardia*: The 2 studies evaluated the frequency of bradycardia, without finding any significant difference between the premedicated and control groups (83, 84).

*Hypotension*: Both studies assessed blood pressure or arterial hypotension. No significant difference was found between the premedicated and control groups (83, 84).

*Pain and comfort*: In both studies, patient comfort during the procedure assessed by the COMFORTneo (85) and R-PIPP (86) scales was significantly improved by premedication.

*Procedure conditions*: The rates of intubation during the procedure were not significantly different between the premedicated and control groups.

*In-hospital mortality and morbidity*: No increase in in-hospital adverse events was observed in the premedicated group in both studies (83, 84).

*Additional information*: Several observational studies have not identified any short-term tolerance issues, but the risk of respiratory depression requiring mechanical ventilation needs to be assessed by other ongoing studies (87). A review of the literature published in 2022, including all types of studies, concluded to an overall effectiveness of premedication before LISA/MIST, without significant risk of poor tolerance (88). Finally, an observational study found a frequent occurrence of poor technical conditions and poor clinical tolerance in case of LISA/MIST without sedo-analgesia (89). No data are available to date on neurodevelopmental follow-up.

**Summary (Moderate LoE)**: There is no doubt that the laryngoscopy required for LISA is as painful and uncomfortable as that required for tracheal intubation. Therefore, appropriate measures should be implemented, although the literature on this topic is still limited. Unlike intubation, the maintenance of effective respiratory activity is essential for this procedure, making it difficult to adjust the level of sedation/analgesia and proscribing the use of any paralytic.

#### 4.3.2. Question 2: Can IV opioids be used as a premedication prior to LISA in neonates?

**Statement**: The experts suggest to consider IV fentanyl as a possible premedication before LISA in neonates (expert opinion).

*Precautions related to gestational age*: The neonates included in the 2 assessed randomized trials evaluating the use of fentanyl had gestational ages from 28^+0^ to 36^+6^ weeks.

*Precautions on hemodynamic status*: No data are available on this point.

**Rationale (Supplementary Table S8)**: Only one randomized trial evaluated the efficacy of a single dose of fentanyl 1 µg/kg IV (*n* = 17) given before surfactant instillation by the LISA method vs. no treatment (*n* = 17), in neonates born between 28 and 33^+6^ weeks (84). A randomized trial evaluated the efficacy of the MIST procedure after administration of fentanyl (1 µg/kg) compared with intubation before intra-tracheal instillation of surfactant in 45 newborns born between 32 and 36^+6^ weeks of gestation with respiratory distress syndrome (90).

*LISA failure*: Failure of the procedure was defined as intubation within 72 h of the procedure and was not significantly increased by fentanyl administration in the randomized trial versus no treatment (84). The rate of intubation within 72 h in the MIST group in the other study was 29% (90).

*Number of attempts*: The number of laryngoscopy attempts to perform LISA was not significantly changed by premedication in these 2 studies (84, 90).

*Duration of the procedure*: These 2 studies did not evaluate the duration of the procedure.

*Hypoxia*: Both studies evaluated the occurrence of desaturations (84, 90). The randomized study comparing fentanyl vs. no treatment did not find a significant difference in the frequency of desaturations (84). In the MIST vs. intubation study, 100% of children in the MIST group had desaturation <80% (90).

*Bradycardia*: Only the randomized study comparing fentanyl vs. no treatment assessed the frequency of bradycardia without finding a significant difference between the premedicated and control groups (84).

*Hypotension*: Only the randomized study comparing fentanyl vs. no treatment assessed blood pressure during the LISA procedure (84). No significant difference was observed between the premedicated and control groups.

*Procedure conditions*: Only the randomized study comparing fentanyl vs. no treatment assessed patient comfort during the procedure using the R-PIPP scale (86). This scale was significantly improved by premedication (84). In the study comparing fentanyl vs. no treatment the rates of intubation during the procedure were not significantly different between the premedicated and control groups (84). In the MIST vs. intubation study, 2 out of 24 (8.3%) cases of chest rigidity were observed in the MIST procedure group requiring intubation during the procedure (90).

*Additional information*: According to several declarative surveys, opiates, and in particular fentanyl, are frequently used in several countries as premedication before LISA (18, 20, 91). A retrospective study reported a rate of chest rigidity or apnea requiring intubation of 5% (5/101) (92).

**Summary (low LoE)**: The use of opioids as premedication before LISA seems feasible in preterm neonates older than 28 weeks of gestation. As with intubation, the use of morphine does not seem appropriate because of its pharmacokinetic characteristics. Fentanyl is the only opioid that has been evaluated in a randomized trial. Nevertheless, the efficacy and safety of opioids in this indication are insufficiently documented. Other premedication modalities should be considered before using opioids.

#### 4.3.3. Question 3: Can IV propofol be used as a premedication prior to LISA in neonates?

**Statement**: Propofol should probably be considered as a possible premedication prior to LISA in neonates (discretionary recommendation).

*Precautions related to gestational age*: The only randomized trial included neonates from 26 to 37 weeks of gestation. Cohorts report the use of propofol in neonates <26 weeks of gestation.

*Precautions on hemodynamic status*: Contraindication in case of hemodynamic instability or hypotension.

**Rationale (Supplementary Table S9)**: Only one randomized study compared propofol (*n* = 42) with no sedation (*n* = 36) for LISA (83).

*LISA failure*: The intubation rate was not significantly increased by propofol (83).

*Number of attempts*: The number of laryngoscopy attempts for LISA was not significantly modified by propofol (83).

*Duration of the procedure*: The duration of the procedure was not significantly modified by propofol (83).

*Hypoxia*: The frequency of desaturations was significantly increased in the propofol group (83).

*Bradycardia*: The frequency of bradycardia was not significantly modified by propofol (83).

*Hypotension*: The mean arterial pressure and the frequency of arterial hypotension were not significantly modified by propofol (83).

*Pain and comfort*: Patient comfort during the procedure, assessed by the COMFORTneo scale (85), was significantly improved by propofol (83).

*In-hospital mortality and morbidity*: No increase in in-hospital adverse events was observed in the propofol group (83).

*Additional information*: The effectiveness of propofol on the comfort of neonates was found in 2 observational studies (93, 94). No long-term follow-up study is available.

**Summary (moderate LoE)**: Propofol appears to be effective on comfort/pain and fairly well tolerated for the LISA procedure, except for the increase in desaturations. Nevertheless, the data are still insufficient to advocate its use with a high level of evidence.

#### 4.3.4. Question 4: Can IV ketamine be used as a premedication in neonates prior to LISA?

**Statement**: The experts suggest that ketamine should be considered as a possible premedication before LISA in neonates (expert opinion).

*Precautions related to gestational age*: Cohort studies report the use of ketamine in premature neonates <30 weeks of gestation.

*Precautions on hemodynamic status*: No specific data available. Ketamine is used in routine practice in older children and adults in case of hemodynamic instability.

**Rationale**: No randomized trial has evaluated ketamine before LISA in neonates. A cohort study compared the use of ketamine (*n* = 52) and propofol (*n* = 62) without demonstrating a significant difference in the need for subsequent intubation up to 2 h after LISA (93). There was also no difference in the frequency of occurrence of hypotension in this cohort comparing ketamine (0.5–1 mg/kg IV) with propofol (1–2 mg/kg). A prospective study evaluated the efficacy and tolerability of ketamine (0.5 mg/kg increments, median cumulative dose 1.5 mg/kg) with atropine (15 μg/kg) in 29 very preterm neonates before LISA (95). Pain scores were mostly low but the rate of respiratory adverse events was quite high with 24% intubation required before LISA. No long-term follow-up studies are available.

**Summary (very low LoE)**: Ketamine appears to be effective on comfort/pain for the LISA procedure. Its respiratory tolerance appears to be dose-dependent. Other sedation-analgesia modalities should be considered before using this molecule.

### 4.4. Premedication before laryngeal mask insertion in neonates

#### 4.4.1. Question 1: Should premedication be performed in neonates before inserting a laryngeal mask except for immediate life-threatening emergencies?

**Statement**: The experts suggest, as for tracheal intubation, to use premedication before the insertion of a laryngeal mask in neonates (expert opinion).

*Precautions related to gestational age*: Precautions related to the limits of the device (rather after 34 weeks of gestation and >1,500 g), then to those of the molecules retained for premedication.

*Precautions on hemodynamic status*: According to the chosen molecules.

**Rationale**: The laryngeal mask is a supra-glottic medical device whose application and maintenance are probably stressful, uncomfortable and are probably accompanied by side effects comparable to those of an awake intubation. A few studies comparing laryngeal mask to tracheal intubation for surfactant instillation (96), or in case of failure of tracheal intubation (97), have shown the feasibility of the procedure during resuscitation in the delivery room. In addition, the laryngeal mask has been included in the ERC and AHA algorithms for the management of neonatal resuscitation (98, 99). There are no studies comparing the use of a laryngeal mask with or without premedication in neonates.

**Summary (very low LoE)**: The current data in the literature are insufficient to recommend a premedication protocol for the application of a laryngeal mask in neonates, but this is widely performed by resuscitation and anesthesia teams who have experience in the use of the laryngeal mask in neonates. The experts therefore propose that a premedication regimen identical to that which would be chosen for tracheal intubation be carried out.

### 4.5. Administration of atropine before upper airways access in neonates

#### 4.5.1. Question 1: Should neonates receive atropine prior to tracheal intubation other than in an immediate life-threatening situation?

**Statement**: The experts suggest that atropine should be administered either preventively, particularly when a depolarizing muscle-blocker is used, or in the event of bradycardia during tracheal intubation outside of an immediate life-threatening emergency (Expert opinion).

*Precautions related to gestational age*: No specific data available.

*Precautions on hemodynamic status*: Probably to be avoided in case of pre-existing tachycardia.

**Rationale**: A randomized, controlled trial compared 3 modalities of premedication: atropine alone (*n* = 10) vs. atropine + pancuronium (*n* = 10) vs. no premedication (*n* = 10) (1). Although it is currently not recommended to perform awake intubation or to use muscle blockers without analgesia or sedation, this study found a smaller decrease in heart rate in the atropine groups. This effect was more pronounced when atropine was combined with a muscle blocker (pancuronium).

*Additional information*: Seven randomized studies evaluated various combinations of opioids and muscle blocker with atropine for tracheal intubation against various comparators: atropine + fentanyl + rocuronium (*n* = 20) vs. atropine + fentanyl (*n* = 24) (48); atropine + fentanyl + mivacurium (*n* = 21) vs. atropine + fentanyl (*n* = 29) (44); atropine + fentanyl + suxamethonium (*n* = 15) vs. atropine + remifentanil (*n* = 15) (46); atropine + morphine + suxamethonium (*n* = 30) vs. propofol (*n* = 33) (45); atropine + morphine + suxamethonium (*n* = 10) vs. no treatment (*n* = 10) (36); atropine + sufentanil + atracurium (*n* = 82) vs. atropine + propofol (*n* = 89) (49); glycopyrrolate + thiopental + remifentanil + suxamethonium (*n* = 17) vs. atropine + morphine (*n* = 17) (47). Bradycardia could occur despite the use of atropine. None of these studies were designed to investigate the effect of atropine alone. No serious adverse effects related to its use in combination with sedative and/or analgesic drugs have been reported. A cohort study including 153 neonates, 79 of whom received atropine prior to critical care intubation, reported no impact on mortality (100).

**Summary (very low LoE)**: There is no argument for or against the routine use of atropine before neonatal intubation. Because of the known vagal hyperreactivity in neonates, experts believe it is reasonable to administer atropine preventively or to prepare atropine for injection if bradycardia occurs during the procedure.

#### 4.5.2. Question 2: Should neonates receive atropine prior to LISA?

**Statement**: The experts suggest that atropine should be administered preventively or in the event of bradycardia in neonates during LISA (Expert opinion).

*Precautions related to gestational age*: No specific data available.

*Precautions on hemodynamic status*: Probably to be avoided in case of pre-existing tachycardia.

**Rationale**: No randomized study has evaluated atropine in neonates prior to LISA. The two randomized studies of premedication before LISA did not include atropine (83, 84). In observational studies, 2 combined atropine with an anesthetic [propofol (*n* = 35) (101) or ketamine (*n* = 29) (95)] and two used an anesthetic without atropine [propofol (*n* = 23) or no treatment (*n* = 23) (94); propofol (*n* = 62) or ketamine (*n* = 52) (93)]. No serious adverse events associated with the use or no-use of atropine were reported in these studies.

**Summary (very low LoE)**: There is no argument for or against the routine use of atropine before LISA. Because of the known vagal hyperreactivity in neonates, the experts believe that it is reasonable to administer atropine preventively or to prepare atropine for injection in case of bradycardia during the procedure.

#### 4.5.3. Question 3: Should neonates receive atropine before a laryngeal mask insertion except in an immediate life-threatening situation?

**Statement**: The experts suggest administering atropine preventively or in the event of bradycardia when a laryngeal mask is inserted, as in the case of tracheal intubation or LISA (Expert opinion).

*Precautions related to gestational age*: No specific data available.

*Precautions on hemodynamic status*: Probably to be avoided in case of pre-existing tachycardia.

**Rationale**: No randomized study has evaluated atropine in neonates prior to laryngeal mask insertion. The experts therefore propose to extrapolate what is done during other laryngoscopies.

**Summary (very low LoE)**: There is no argument for or against the routine use of atropine before laryngeal mask insertion in neonates. Because of the known vagal hyperreactivity in neonates, the experts believe that it is reasonable to administer atropine preventively or to prepare atropine for injection in case of bradycardia during the procedure.

## 5. Doses

Table 3 summarizes the recommended doses for the vagolytic agent, muscle-blockers and opioids discussed in this document, according to indication and gestational age. Table 4 summarizes the recommended doses for the sedatives and anesthetics discussed in this document, according to indication and gestational age. These data are based on the literature (12, 13, 36, 44–49, 51–53, 68, 69, 71, 81, 83, 84, 90, 94, 101–110). Whenever possible, drugs should be started at a low dose and titrated by small increments so that the minimal effective dose can be administered.

**Table 3:** Proposed doses for possible vagolytics, muscle blockers and opioids for premedication before laryngoscopy.

Molecule	Class	Onset delay	Duration of action	Procedures	Doses according to corrected gestational age
					<28 weeks	28–32 weeks	>32 weeks
**Vagolytic**							
Atropine	Vagolytic	1 min	2 h	Intubation	10–20 µg/kg
				LISA/MIST	
				Laryngeal mask^a^	
**Muscle blockers**							
Succinylcholine (Suxamethonium)	Muscle blocker (depolarizing)	1 min	3–10 min	Intubation	2 mg/kg
				LISA/MIST	Not recommended
				Laryngeal mask^a^	N/A	2 mg/kg
Atracurium	Muscle blocker (non-depolarizing)	2 min	15–30 min	Intubation	0.3 mg/kg	0.5 mg/kg
				LISA/MIST	Not recommended
				Laryngeal mask^a^	0.3 mg/kg	0.5 mg/kg
Mivacurium	Muscle blocker (non-depolarizing)	1.5–2 min	15–30 min	Intubation	0.2 mg/kg
				LISA/MIST	Not recommended
				Laryngeal mask^a^	N/A	0.2 mg/kg
Rocuronium	Muscle blocker (non-depolarizing)	1–3 min	40–60 min	Intubation	0.5 mg/kg
				LISA/MIST	Not recommended
				Laryngeal mask^a^	N/A	0.5 mg/kg
**Opioids**							
Fentanyl	Opioid	1.5 min	T½: 9.5 h	Intubation	1–2 µg/kg	2–3 µg/kg	3–4 µg/kg
				LISA/MIST	?	1 µg/kg
				Laryngeal mask^a^	N/A	2–3 µg/kg	3–4 µg/kg
Sufentanil	Opioid	30–60 s	15–20 min	Intubation	0.1–0.2 μg/kg	0.2–0.3 µg/kg
				LISA/MIST	?
				Laryngeal mask^a^	N/A	0.2–0.3 µg/kg	

LISA, less invasive surfactant administration; MIST, minimally invasive surfactant treatment; N/A, not applicable; IV, intravenous; IN, intranasal; T½, half-life.

^a^ In the absence of published data on premedication for laryngeal mask placement in neonates, these proposals are extrapolations of those made for tracheal intubation.

**Table 4:** Proposed doses for possible sedatives and anesthetics for premedication before laryngoscopy.

Molecule	Class	Onset delay	Duration of action	Procedures	Doses according to corrected gestational age
					<28 weeks	28–32 weeks	>32 weeks
Ketamine	Sedative/anesthetic	IV 1–5 min	IV = 10–20 min	Intubation	1–2 mg/kg IV 2–4 mg/kg IN	2–3 mg/kg IV 2– 4 mg/kg IN	3–5 mg/kg IV 2– 4 mg/kg IN
				LISA/MIST	Titration by increments of 0.5 mg/kg Maximum dose 1 mg/kg	Titration by increments of 0.5 mg/kg Maximum dose 1.5 mg/kg	Titration by increments of 0.5 mg/kg Maximum dose 2 mg/kg
				Laryngeal mask^a^	N/A	2–3 mg/kg IV	3–5 mg/kg IV
Propofol	Anesthetic	1 min	T½: 13 min	Intubation	Titration by increments of 1 mg/kg Maximum dose 2 mg/kg	Titration with 1st dose 2 mg/kg If reinjection: 1 mg/kg Maximum dose 4 mg/kg	Titration with 1st dose 2 mg/kg If reinjection: 1 mg/kg Maximum dose 5 mg/kg
				LISA/MIST	Titration by increments of 0.5–1 mg/kg Maximum dose 2 mg/kg	Titration by increments of 0.5–1 mg/kg Maximum dose 2 mg/kg	Titration by increments of 1 mg/kg Maximum dose 2 mg/kg
				Laryngeal mask^a^	N/A	Titration with 1st dose 2 mg/kg If reinjection: 1 mg/kg Maximum dose 4 mg/kg	Titration with 1st dose 2 mg/kg If reinjection: 1 mg/kg Maximum dose 5 mg/kg
Midazolam	Benzodiazepine/hypnotic	1–2 min (if IN: 5 min)	T½: 6.3 h	Intubation	IV route not recommended 100–200 µg/kg IN	50 µg/kg IV 100–200 µg/kg IN	50–200 µg/kg IV 100–200 µg/kg IN
				LISA/MIST	?	?	?
				Laryngeal mask^a^	N/A	50 µg/kg	50–200 µg/kg

LISA, less invasive surfactant administration; MIST, minimally invasive surfactant treatment; N/A, not applicable; IV, intravenous; IN, intranasal; T½, half-life.

^a^ In the absence of published data on premedication for laryngeal mask placement in neonates, these proposals are extrapolations of those made for tracheal intubation.

## 6. Perspectives

These good practice statements for premedication before laryngoscopy in neonates are a synthesis of the current literature and should evolve according to future data. In a perspective of constant improvement of practices, other objective parameters should be taken into account that are currently rarely reported in the literature: the conditions of intubation and the pain provoked by the procedure, as well as the long-term outcome of these children. The Acute Neonatal Pain (ANP) (65) or Faceless Acute Neonatal Scale (FANS) (43) can be used to estimate pain. The conditions of intubation, evaluating the infant's tone and reactivity on the one hand, and the relaxation of the jaw, vocal cords opening and thoracic movements during laryngoscopy on the other hand, would make it possible to appreciate the effectiveness or not of the molecules and doses used. The TRACHEA score (14), the Intubation Readiness Score (IRS) (111) and the Viby-Mogensen score (112) are useful tools to measure the depth of sedation of the newborn. Finally, rigorous long-term follow-up of neonates included in research protocols evaluating premedication should address concerns about the neurodevelopmental effects of sedative and analgesic molecules used (87).

## 7. Conclusion

**Figure 2:**
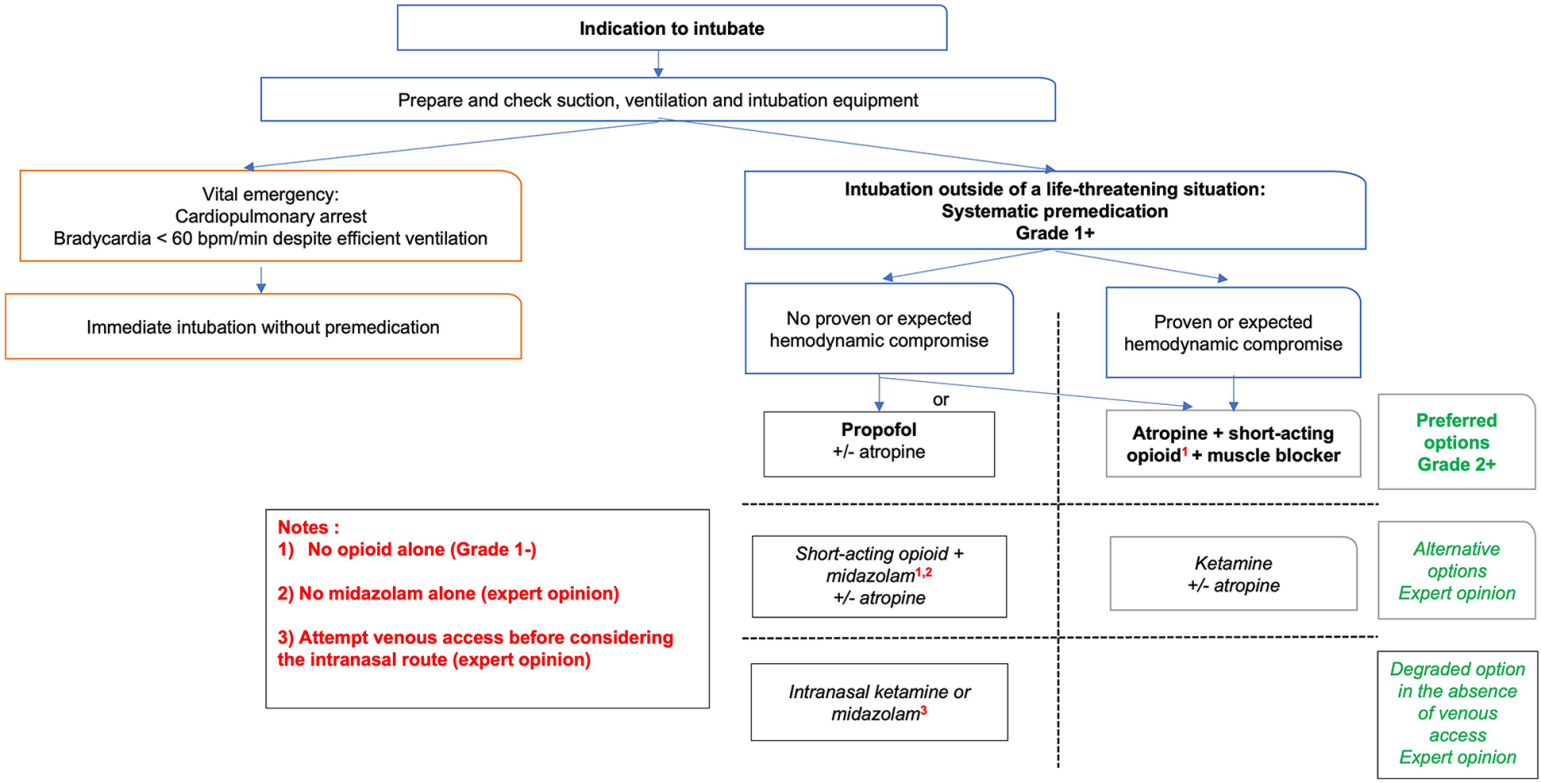
Decision chart for premedication before tracheal intubation in neonates.

Current knowledge on the physiological effects of awake laryngoscopy in neonates supports routine use of premedication before performing this procedure, whether for intubation (Grade 1+) or LISA (Grade 2+). Evidence regarding premedication for laryngeal mask insertion or routine use of atropine before laryngoscopy is lacking. The summary work carried out has made it possible to formulate good practice statements including premedication with propofol in the absence of proven or expected hemodynamic disorders (Grade 2+) or a combination of opioid + muscle blocker (Grade 2+) for tracheal intubation; and premedication with propofol for LISA (Grade 2+). Figure 2 summarizes these best practice statement for tracheal intubation and includes second-line options based on expert opinion. New research knowledge will allow for future updates of these statements.
