# Supplementary material for: Premedication before laryngoscopy in neonates: Evidence-based statement from the French society of neonatology (SFN)
Source: Front Pediatr. 2023 Jan 4;10:1075184. doi: 10.3389/fped.2022.1075184 (PMC9846576; doi:10.3389/fped.2022.1075184)
Supplement: Supplementary file 1 [file Table1.docx]

Premedication before laryngoscopy in neonates: evidence-based statement from the French Society of Neonatology (SFN)- Supplementary material

**Literature search performed in September 2021, with no time limitation – articles in English or French language only.**

**MeSH terms used for the literature search in Pubmed, web of science, scopus, Mbase:**

("premature*"[All Fields] OR "newborn*"[All Fields]) AND ("esketamine"[Supplementary Concept] OR "esketamine"[All Fields] OR "ketamine"[All Fields] OR "ketamine"[MeSH Terms] OR "ketamin"[All Fields] OR "ketamine s"[All Fields] OR "ketamines"[All Fields] OR ("midazolam"[MeSH Terms] OR "midazolam"[All Fields] OR "midazolam s"[All Fields] OR "midazolame"[All Fields]) OR ("morphine derivatives"[MeSH Terms] OR ("morphine"[All Fields] AND "derivatives"[All Fields]) OR "morphine derivatives"[All Fields] OR "morphines"[All Fields] OR "morphine"[MeSH Terms] OR "morphine"[All Fields] OR "morphin"[All Fields] OR "morphine s"[All Fields] OR "morphinic"[All Fields]) OR ("curare"[MeSH Terms] OR "curare"[All Fields] OR "curares"[All Fields] OR "curarization"[All Fields] OR "curarized"[All Fields] OR "curarizing"[All Fields]) OR ("atropine"[MeSH Terms] OR "atropine"[All Fields] OR "atropin"[All Fields] OR "atropinization"[All Fields] OR "atropinized"[All Fields] OR "hyoscyamine"[MeSH Terms] OR "hyoscyamine"[All Fields]) OR ("nitrogen oxides"[MeSH Terms] OR ("nitrogen"[All Fields] AND "oxides"[All Fields]) OR "nitrogen oxides"[All Fields]) OR ("propofol"[MeSH Terms] OR "propofol"[All Fields] OR "propofol s"[All Fields]) OR ("clonidine"[MeSH Terms] OR "clonidine"[All Fields] OR "clonidin"[All Fields] OR "clonidine s"[All Fields]) OR ("dexmedetomidine"[MeSH Terms] OR "dexmedetomidine"[All Fields] OR "dexmedetomidine s"[All Fields])) AND "intub*"[All Fields]

The same search was performed with the same key words without using MeSH terms.

This search resulted in 451 publications that were screened and selected by all the experts.

| **Supplementary Table 1: Randomized trials comparing premedication versus awake intubation for tracheal intubation in neonates** | | | | | | | | | | | | | | | | |
| --- | --- | --- | --- | --- | --- | --- | --- | --- | --- | --- | --- | --- | --- | --- | --- | --- |
| **General** | | | | | **Quality** | | | | | **Patient** | | **Effect** | | | **Synthesis** | |
| **Outcome** | **Intervention** | **Author** | **Year** | **Journal** | **Type** | **Consistency (I²)** | **Direct effect** | **Precision of CI** | **Other**  **bias?** | **Studied group** | **Control group** | **P-value** | **Relative risk** | **Absolute difference** | **Quality** | **Importance** |
| **Number of attempts for intubation** | | | | | | | | | | | | | | | | |
| N median [IQR] | Atropine + morphine + suxamethonium (n=10)  vs no medication (n=10) | Oei | 2002 | J Paediatr Child Health | Open RCT | Monocentric  Preterm and term  Intervention:  29 (25-39)^a^ wks  Control:  30.5 (25-40)^a^ wks | + | - | No | 1 (1-3) | 2 (1-6) | **P=0.035** | - | - | Moderate | High |
| N median [IQR] | Morphine (n=17)  vs placebo (n=17) | Lemyre | 2004 | BMC Pediatr | Double-blind RCT | Monocentric  Preterm and term  Morphine:  28 (26-33) ^b^ wks  Placebo:  27 (26-30) ^b^ wks | + | - | No | 2 (1-3.5) | 1 (1-2.5) | P=0.34 |  | - | High | High |
| Success rate at 1^st^ attempt. % | Sevoflurane (n=24)  vs no medication (n=18) | Hassid | 2007 | Paediatr Anaesth | Open RCT | Monocentric  Preterm and term  Sevoflurane:  31 (4.8)^c^ wks  Control:  29.8 (3.8)^c^ wks  Several attempts for one patient taken into account for analysis | + | - | No | 25%  N not available | 39%  N not available | NS  P not provided | - | - | Low | Moderate |
| Success rate at 1^st^ attempt. n/N (%) | Atropine + remifentanil (n=20)  vs atropine (n=20) | Badiee | 2013 | J Res Pharm Pract | Double-blind RCT | Bi-centric  Preterm  Remifentanil: 30.6 (2.8)^c^ wks  Control:  31.7 (3.5)^c^ wks  Exclusion if ≥ 3 attempts | - | 95% | Inadequate randomization | 17/20 (85) | 16/20 (80) | P=0.67 | 1.1*  (0.8-1.4) | +5 %*  (-18 to 28) | Very Low | Low |
| Success rate at 1^st^ attempt, % | Atropine + midazolam (n=40)  vs atropine + placebo (n=40) | Badiee | 2021 | J Res Med Sci | Double-blind RCT | Monocentric  Preterm  Midazolam:  31.8 (2.6)^c^ wks  Control:  29.8 (0.3)^c^ wks  Exclusion if ≥ 2 attempts | + | - | Ambiguous randomization | 92.5%  N not available | 66.7%  N not available | **P=0.004** | - | - | Low | Low |
| **Duration of intubation** | | | | | | | | | | | | | | | | |
| Mean  (+/- SD) | Thiopental (n =14)  vs placebo (n =13) | Bhutada | 2000 | Arch Dis Child Fetal Neonatal Ed | Open RCT | Monocentric  Term  Thiopental:  39.6 (1.2)^c^ wks  Control:  39.2 (1.4)^c^ wks | + | 95% | No | 2.7 (0.4) min | 5.08 (1.1) min | **P<0.04** |  | -2.4 min*  (-3.0 to -1.7) | Moderate | High |
| Median (IQR) | Atropine + morphine + suxamethonium (n=10)  vs no medication (n=10) | Oei | 2002 | J Paediatr Child Health | Open RCT | Monocentric  Preterm and term  Intervention: 29 (25-39)^a^ wks  Control:  30.5 (25-40)^a^ wks | + | - | No | 60 s  (40-1095) | 595 s  (60-1820) | **P=0.02** |  | - | Moderate | High |
| Median (IQR) | Morphine (n=17) vs placebo (n=17) | Lemyre | 2004 | BMC Pediatr | Double-blind RCT | Monocentric  Preterm and term  Morphine:  28 (26-33)^b^ wks  Placebo:  27 (26-30)^b^ wks | + | - | No | 271 s  (57.5-418.5) | 94 s  (62.0-215.5) | P=0.27 | - | - | High | High |
| Mean  (+/- SD) | Atropine + remifentanil (n=20)  vs atropine (n=20) | Badiee | 2013 | J Res Pharm Pract | Double-blind RCT | Bi-centric  Preterm  Remifentanil: 30.6 (2.8)^c^ wks  Control:  31.7 (3.5)^c^ wks  Exclusion if ≥ 3 attempts | - | 95% | Inadequate randomization | 20.8 (6.0) s | 22.8 (7.3) s | P=0.34 | - | -2 s*  (-6.3 to 2.3) | Very Low | Moderate |
| Mean  (+/- SD) | Atropine + midazolam (n=40)  vs atropine + placebo (n=40) | Badiee | 2021 | J Res Med Sci | Double-blind RCT | Monocentric  Preterm  Midazolam:  31.8 (2.6)^c^ wks  Control:  29.8 (0.3)^c^ wks  Exclusion if ≥ 2 attempts | + | 95% | Ambiguous randomization | 18.8 (4.8) s | 23.5 (6.7) s | **P=0.001** | - | -4.7 s*  (-7.3 to -2.1) | Low | Moderate |
| **Hypoxia** | | | | | | | | | | | | | | | | |
| Delta SpO_2_, mean  (+ /- SD) | Thiopental (n =14)  vs placebo (n =13) | Bhutada | 2000 | Arch Dis Child Fetal Neonatal Ed | Open RCT | Monocentric  Term  Thiopental:  39.6 (1.2)^c^ wks  Control:  39.2 (1.4)^c^ wks | + | 95% | No | -11.2 (3.7) % | -14.6 (5.3) % | P=0.18 |  | +3.4 %*  (-0.2 to 7) | Moderate | High |
| Nadir SpO_2_  mean  (+ /- SD) | Atropine + morphine + suxamethonium (n=10)  vs no medication (n=10) | Oei | 2002 | J Paediatr Child Health | Open RCT | Monocentric  Preterm and term  Intervention:  29 (25-39)^a^ wks  Control:  30.5 (25-40)^a^ wks | + | 95% | No | 60 ± 20 % | 58 ± 22 % | P=0.271 | - | +2 %*  (-18 to 22) | Moderate | High |
| SpO_2_<85%, n/N (%) | Morphine (n=17)  vs placebo (n=17) | Lemyre | 2004 | BMC Pediatr | Double-blind RCT | Monocentric  Preterm and term  Morphine:  28 (26-33) wks^b^  Placebo:  27 (26-30) wks^b^ | + | 95% | No | 17/17 (100) | 14/17 (82) | P=0.23* | 1.2*  (0.97-1.5) | +18 %*  (-0.5 to 36) | High | High |
| SpO_2_<85%  > 30 s, n/N (%) | Sevoflurane (n=24)  vs no medication (n=18) | Hassid | 2007 | Paediatr Anaesth | Open RCT | Monocentric  Preterm and term  Sevoflurane:  31 (4.8)^c^ wks  Control:  29.8 (3.8)^c^ wks  Several attempts for one patient taken into account for analysis | + | 95% | No | 9/24 (37.5) | 8/18 (44.4) | NS  P not provided | 0.8*  (0.4 – 1.8) | -6.9 %*  (-37 to 23) | Low | High |
| SpO_2_<70%, n/N (%) | Atropine + remifentanil (n=20)  vs atropine (n=20) | Badiee | 2013 | J Res Pharm Pract | Double-blind RCT | Bi-centric  Preterm  Remifentanil: 30.6 (2.8)^c^ wks  Control:  31.7 (3.5)^c^ wks  Exclusion if ≥ 3 attempts | - | 95% | Inadequate randomization | 4/20 (20) | 1/20 (5) | P=0.17 | 4*  (0.5 – 33) | +15 %*  ( -5 to 35) | Very Low | Moderate |
| SpO_2_<70%, n/N (%) | Atropine + midazolam (n=40)  vs atropine + placebo (n=40) | Badiee | 2021 | J Res Med Sci | Double-blind RCT | Monocentric  Preterm  Midazolam:  31.8 (2.6)^c^ wks  Control:  29.8 (0.3)^c^ wks  Exclusion if ≥ 2 attempts | + | 95% | Ambiguous randomization | 2/40 (5%) | 22/40 (55%) | **P=10^-4^** | 0.09*  (0.02-0.36) | -50 %*  (-67 to -33) | Low | Moderate |
| **Bradycardia** | | | | | | | | | | | | | | | | |
| HR<90/min, n/N (%) | Morphine (n=17) vs placebo (n=17) | Lemyre | 2004 | BMC Pediatr | Double-blind RCT | Monocentric  Preterm and term  Morphine:  28 (26-33)^b^ wks  Placebo:  27 (26-30)^b^ wks | + | 95% | No | 16/17 (94.1) | 12/17 (70.6) | P=0.18 | 1.3*  (0.96 –1.9) | +24 %*  (-0.8 to 48) | High | High |
| HR<100/min, n/N (%) | Sevoflurane (n=24) vs no medication (n=18) | Hassid | 2007 | Paediatr Anaesth | Open RCT | Monocentric  Preterm and term  Sevoflurane:  31 (4.8)^c^ wks  Control:  29.8 (3.8)^c^ wks  Several attempts for one patient taken into account for analysis | + | 95% | No | 2/24 (8.3) | 8/18 (44.4) | **P=0.01** | 0.2*  (0.05 –0.8) | -36 %*  (-62 to -11) | Low | High |
| **Hypotension** | | | | | | | | | | | | | | | | |
| Delta BP, mean (+/- SD) | Thiopental (n =14)  vs placebo (n =13) | Bhutada | 2000 | Arch Dis Child Fetal Neonatal Ed | Open RCT | Monocentric  Term  Thiopental:  39.6 (1.2)^c^ wks  Control:  39.2 (1.4)^c^ wks | + | 95% | No | -2.9 (1.8) mmHg | +4.4 (1.1) mmHg | **P<0.002** | - | -7.3 mmHg* (-8.5 to -6.1) | Moderate | Moderate |
| Decrease MBP > 30%, n/N (%) | Sevoflurane (n=24)  vs no medication (n=18) | Hassid | 2007 | Paediatr Anaesth | Open RCT | Monocentric  Preterm and term  Sevoflurane:  31 (4.8)^c^ wks  Control:  29.8 (3.8)^c^ wks  Several attempts for one patient taken into account for analysis | + | 95% | No | 9/24 (37.5) | 6/16 (37.5) | NS  P not provided | 1.0*  (0.4 – 2.3) | 0 %*  (-31 to 31) | Low | Moderate |
| MBP 5 min after intubation | Atropine + remifentanil (n=20)  vs atropine (n=20) | Badiee | 2013 | J Res Pharm Pract | Double-blind RCT | Bi-centric  Preterm  Remifentanil: 30.6 (2.8)^c^ wks  Control:  31.7 (3.5)^c^ wks  Exclusion if ≥ 3 attempts | + | - | Inadequate randomization | 43.6 | 43.0 | - | - | - | Very Low | Low |
| **Pain and comfort** | | | | | | | | | | | | | | | | |
| PIPP score, mean (+/-SD) | Atropine + remifentanil (n=20)  vs atropine (n=20) | Badiee | 2013 | J Res Pharm Pract | Double-blind RCT | Bi-centric  Preterm  Remifentanil: 30.6 (2.8)^c^ wks  Control:  31.7 (3.5)^c^ wks  Exclusion if ≥ 3 attempts | + | 95% | Inadequate randomization | 7.5 (1.4) | 15.1 (1.6) | **P<0.001** | - | -7.6*  (-8.6 to -6.6) | Very Low | High |
| PIPP score after intubation, mean (+/- SD) | Atropine + midazolam (n=40)  vs atropine + placebo (n=40) | Badiee | 2021 | J Res Med Sci | Double-blind RCT | Monocentric  Preterm  Midazolam:  31.8 (2.6)^c^ wks  Control:  29.8 (0.3)^c^ wks  Exclusion if ≥ 2 attempts | + | 95% | Ambiguous randomization | 5.2 (2.06) | 12.9 (2.9) | **P=10^-4^** | - | -7.7*  (-8.8 to -6.6) | Very Low | Moderate |
| FANS score after intubation, mean (+/- SD) | Atropine + midazolam (n=40)  vs atropine + placebo (n=40) | Badiee | 2021 | J Res Med Sci | Double-blind RCT | Monocentric  Preterm  Midazolam:  31.8 (2.6)^c^ wks  Control:  29.8 (0.3)^c^ wks  Exclusion if ≥ 2 attempts | + | 95% | Ambiguous randomization | 0.68 (1.7) | 6.1 (1.5) | **P=10^-4^** | - | -5.4*  (-6.1 to -4.7) | Very Low | Moderate |
| **Intubation conditions** | | | | | | | | | | | | | | | | |
| No movement, n/N (%) | Sevoflurane (n=24)  vs no medication (n=18) | Hassid | 2007 | Paediatr Anaesth | Open RCT | Monocentric  Preterm and term  Sevoflurane:  31 (4.8)^c^ wks  Control:  29.8 (3.8)^c^ wks  Several attempts for one patient taken into account for analysis | + | 95% | No | 21/22 (95.5) | 5/18 (27.8) | **P<0.05** | 3.4*  (1.6 – 7.3) | +67.7 %*  (45.2 to 90.1) | Low | High |
| Good glottis visualization, n/N (%) | Sevoflurane (n=24)  vs no medication (n=18) | Hassid | 2007 | Paediatr Anaesth | Open RCT | Monocentric  Preterm and term  Sevoflurane:  31 (4.8)^c^ wks  Control:  29.8 (3.8)^c^ wks  Several attempts for one patient taken into account for analysis | + | 95% | No | 16/22 (72.7) | 6/18 (33.3) | **P=0.013** | 2.2*  (1.1 – 4.4) | +39 %*  (11 to 68) | Low | High |
| Opened vocal cords, n/N (%) | Sevoflurane (n=24)  vs no medication (n=18) | Hassid | 2007 | Paediatr Anaesth | Open RCT | Monocentric  Preterm and term  Sevoflurane:  31 (4.8)^c^ wks  Control:  29.8 (3.8)^c^ wks  Several attempts for one patient taken into account for analysis | + | 95% | No | 15/22 (68.2) | 9/18 (50) | NS  P not provided | 1.4*  (0.8 – 2.3) | +18 %*  (-12 to 48) | Low | High |
| Excellent conditions (Hans-Cooper), n/N (%) | Atropine + midazolam (n=40)  vs atropine + placebo (n=40) | Badiee | 2021 | J Res Med Sci | Double-blind RCT | Monocentric  Preterm  Midazolam:  31.8 (2.6)^c^ wks  Control:  29.8 (0.3)^c^ wks  Exclusion if ≥ 2 attempts | + | 95% | Ambiguous randomization | 33/40 (82.5) | 0/40 (0) | **P=10^-4^** | - | +82.5 %*  (71 to 94) | Very Low | Low |
| * Test performed by the working group  ^a^Mean (range)  ^b^Median (Interquartile range)  ^c^Mean (SD)  Abbreviations: CI, confidence interval; NS, not significant; RCT, randomized controlled trial; wks, weeks of gestation; SpO_2_, oxygen saturation; HR, heart rate; BP, blood pressure; MBP, mean blood pressure; PIPP, premature infant pain profile; FANS, faceless acute neonatal pain score | | | | | | | | | | | | | | | | |

| **Supplementary Table 2: Randomized trials comparing premedication with a combination of an IV opioid and a muscle blocker versus another regimen for tracheal intubation in neonates** | | | | | | | | | | | | | | | | |
| --- | --- | --- | --- | --- | --- | --- | --- | --- | --- | --- | --- | --- | --- | --- | --- | --- |
| **General** | | | | | **Quality** | | | | | **Patient** | | **Effect** | | | **Synthesis** | |
| **Outcome** | **Intervention** | **Author** | **Year** | **Journal** | **Type** | **Consistency (I²)** | **Direct effect** | **Precision of CI** | **Other**  **bias?** | **Studied group** | **Control group** | **P-value** | **Relative risk** | **Absolute difference** | **Quality** | **Importance** |
| **Number of attempts for intubation** | | | | | | | | | | | | | | | | |
| N median [IQR] | Atropine + morphine + suxamethonium (n=10)  vs no medication (n=10) | Oei | 2002 | J Paediatr Child Health | Open RCT | Monocentric  Preterm and term  Intervention:  29 (25-39)^a^ wks  Control:  30.5 (25-40)^a^ wks | + | - | No | 1 [1-3] | 2 [1-6] | **P=0.035** | - | - | Moderate | High |
| N median [IQR] | Fentanyl + mivacurium + atropine (n=21)  vs fentanyl + atropine (n=20) | Roberts | 2006 | Pediatrics | Open RCT | Bi-centric  Preterm and term  Intervention: 29.7 (24-38)^a^  wks  Control:  30.2 (24-42)^a^ wks | + | - | No | 2 [1-4] | 4 [1-9] | P=0.066 | - | - | Moderate | High |
| ≤ 2 attempts before successful intubation, n/N (%) | Fentanyl + mivacurium + atropine (n=21)  vs fentanyl + atropine (n=20) | Roberts | 2006 | Pediatrics | Open RCT | Bi-centric  Preterm and term  Intervention: 29.7 (24-38)^a^  wks  Control:  30.2 (24-42)^a^ wks | + | 95% | No | 15/21 (71) | 7/20 (35) | **P=0.019** | 2*  (1.1 – 3.9) | +36 %*  (8 to 65) | Moderate | High |
| N median [IQR] | Morphine + atropine + suxamethonium (n=30)  vs propofol (n=33) | Ghanta | 2007 | Pediatrics | Open RCT | Monocentric  Preterm and term  Morphine+sux: 28 (25-31)^b^ wks  Propofol:  27 (25-30)^b^ wks | + | - | No | 2 [1-3] | 1 [1-2] | P=0.08 | - | - | Moderate | High |
| Success rate at 1^st^ attempt, n/N (%) | Fentanyl + suxamethonium + atropine (n=15)  vs atropine + remifentanil (n=15) | Choong | 2010 | Arch Dis Child Fetal Neonatal Ed | Double-blind RCT | Monocentric  Preterm and term  Fentanyl+sux: 27.1 (25.6-28.7)^b^ wks  Remifentanil:  28 (25-30)^b^ wks | + | 95% | No | 6/15 (40) | 9/15 (60) | P=0.47 | 0.7*  (0.3-1.4) | -20 %*  (-55 to 15) | High | High |
| N mean (SD) | Fentanyl + suxamethonium + atropine (n=15)  vs atropine + remifentanil (n=15) | Choong | 2010 | Arch Dis Child Fetal Neonatal Ed | Double-blind RCT | Monocentric  Preterm and term  Fentanyl+sux: 27.1 (25.6-28.7)^b^ wks  Remifentanil:  28 (25-30)^b^ wks | + | 95% | No | 1.8 (0.8) | 1.7 (0.9) | P=0.67 | - | +0.1*  (-0.5 to 0.7) | High | High |
| N median [IQR] | Glycopyrrolate + thiopental + suxamethonium + remifentanil (n=17)  vs atropine + morphine (n=17) | Norman | 2011 | J Pediatr | Double-blind RCT | Monocentric  Preterm  Intervention:  27 (25.6-28.5)^b^ wks  Morphine:  26.6 (25.1-28.7)^b^ wks | + | - | No | 1 (1-1.5) | 1 (1-2) | NS  P not provided | - | - | High | Mean |
| Success rate at 1^st^ attempt, n/N (%) | Fentanyl + rocuronium + atropine (n=20)  vs fentanyl + atropine (n=24) | Feltman | 2011 | J Perinatol | Open RCT | Monocentric  Preterm<36 wks  Several attempts for one patient taken into account for analysis | + | 95% | No | 7/20 (35) | 2/24 (8) | P=0.057 | 4.2*  (0.98 – 18) | +27 %*  (3 to 50) | Low | Mean |
| Success rate at 1^st^ attempt, n/N (%) | Sufentanil + atracurium + atropine (n=82)  vs propofol + atropine (n=89) | Durrmeyer | 2018 | JAMA | Double-blind RCT | Multicentric  Preterm and term  Sufentanil+atrac: 29 (26-32)^b^ wks  Propofol:  30 (28-34)^b^ wks | + | 95% | Premature stop of inclusions | 47/81 (58.0) | 41/87 (47.1) | P=0.37 | 1.2*  (0.9 – 1.6) | +11 %*  (-4 to 26) | High | High |
| N median [IQR] | Sufentanil + atracurium + atropine (n=82)  vs propofol + atropine (n=89) | Durrmeyer | 2018 | JAMA | Double-blind RCT | Multicentric  Preterm and term  Sufentanil+atrac: 29 (26-32)^b^ wks  Propofol:  30 (28-34)^b^ wks | + | 95% | Premature stop of inclusions | 2 [1-2] | 1 [1-2] | P=0.36 | - | 0 | High | High |
| **Duration of intubation** | | | | | | | | | | | | | | | | |
| Median [IQR] | Morphine + atropine + suxamethonium (n=10)  vs no medication (n=10) | Oei | 2002 | J Paediatr Child Health | Open RCT | Monocentric  Preterm and term  Intervention:  29 (25-39)^a^ wks  Control:  30.5 (25-40)^a^ wks | + | - | No | 60 s  [60-244] | 595 s  [209-846[ | **P=0.02** | - | - | Moderate | High |
| Mean | Fentanyl + mivacurium + atropine (n=21)  vs fentanyl + atropine (n=20) | Roberts | 2006 | Pediatrics | Open RCT | Bi-centric  Preterm and term  Intervention: 29.7 (24-38)^a^ wks  Control:  30.2 (24-42)^a^ wks | + | - | No | 144 s | 472 s | **P=0.003** | - | - | Moderate | High |
| Median [IQR] | Morphine + atropine + suxamethonium (n=30)  vs propofol (n=33) | Ghanta | 2007 | Pediatrics | Open RCT | Monocentric  Preterm and term  Morphine+sux: 28 (25-31)^b^ wks  Propofol :  27 (25-30)^b^ wks | + | - | No | 260 s  [60-435] | 120 s  [60-180] | **P=0.007** | - | - | Moderate | High |
| Median [IQR] | Fentanyl + suxamethonium + atropine (n=15)  Vs atropine + remifentanil (n=15) | Choong | 2010 | Arch Dis Child Fetal Neonatal Ed | Double-blind RCT | Monocentric  Preterm and term  Fentanyl+sux: 27.1 (25.6-28.7)^b^ wks  Remifentanil:  28 (25-30)^b^ wks | + | - | No | 156 s  [46-395] | 247 s  [48-349] | P=0.88 | - | - | High | High |
| Median [IQR] | Glycopyrrolate + thiopental + suxamethonium + remifentanil (n=17) vs atropine + morphine (n=17) | Norman | 2011 | J Pediatr | Double-blind RCT | Monocentric  Preterm  Intervention:  27 (25.6-28.5)^b^ wks  Morphine:  26.6 (25.1-28.7)^b^ wks | + | - | No | 45 s  [35-154] | 97 s  [49-365] | **P=0.031** | - | - | High | High |
| Median [IQR] | Sufentanil + atracurium + atropine (n=82)  vs propofol + atropine (n=89) | Durrmeyer | 2018 | JAMA | Double-blind RCT | Multicentric  Preterm and term  Sufentanil+atrac: 29 (26-32)^b^ wks  Propofol:  30 (28-34)^b^ wks | + | 95% | Premature stop of inclusions | 3.5 min  [1.3- 6.0] | 6.0 min  [2.8- 9.1] | **P=0.03** | - | - 1.7 min  (-0.6 to -3.0) | High | High |
| **Hypoxia** | | | | | | | | | | | | | | | | |
| Nadir SpO_2_  Mean (+/- SD) | Atropine + morphine + suxamethonium (n=10)  vs no medication (n=10) | Oei | 2002 | J Paediatr Child Health | Open RCT | Monocentric  Preterm and term  Intervention:  29 (25-39)^a^ wks  Control:  30.5 (25-40)^a^ wks | + | 95% | No | 60 (20) % | 58 (22) % | P=0.271 | - | +2 %*  (-18 to 22) | Moderate | High |
| SpO_2_≤75%  >30 s, n/N (%) | Fentanyl + mivacurium + atropine (n=21)  vs fentanyl + atropine (n=20) | Roberts | 2006 | Pediatrics | Open RCT | Bi-centric  Preterm and term  Intervention: 29.7 (24-38)^a^ wks  Control:  30.2 (24-42)^a^ wks | + | 95% | No | 6/21 (29) | 11/20 (55) | P=0.09 | 0.5*  (0.2-1.1) | -26 %*  (-56 to -2.7) | Moderate | High |
| SpO_2_ Median [IQR] | Morphine + atropine + suxamethonium (n=30)  vs propofol (n=33) | Ghanta | 2007 | Pediatrics | Open RCT | Monocentric  Preterm and term  Morphine+sux: 28 (25-31)^b^ wks  Propofol:  27 (25-30)^b^ wks | + | 95% | No | 60 [43-82] | 80 [67-88] | **P=0.019** | - | - | Moderate | High |
| Delta SpO_2_, mean  (+ /- SD) | Fentanyl + suxamethonium + atropine (n=15)  vs atropine + remifentanil (n=15) | Choong | 2010 | Arch Dis Child Fetal Neonatal Ed | Double-blind RCT | Monocentric  Preterm and term  Fentanyl+sux: 27.1 (25.6-28.7)^b^ wks  Remifentanil:  28 (25-30)^b^ wks | + | 95% | No | - 47 (25) % | - 55 (27) % | P=0.42 | - | +8 %*  (-11 to 27) | High | High |
| SpO_2_ mean | Glycopyrrolate + thiopental + suxamethonium + remifentanil (n=17) vs atropine + morphine (n=17) | Norman | 2011 | J Pediatr | Double-blind RCT | Monocentric  Preterm  Intervention:  27 (25.6-28.5)^b^ wks  Morphine:  26.6 (25.1-28.7)^b^ wks | + | - | No | Data not available | Data not available | NS  P not provided | - | - | High | Moderate |
| SpO_2_<80%  > 60 s, n/N (%) | Sufentanil + atracurium + atropine (n=82)  vs propofol + atropine (n=89) | Durrmeyer | 2018 | JAMA | Double-blind RCT | Multicentric  Preterm and term  Sufentanil+atrac: 29 (26-32)^b^ wks  Propofol:  30 (28-34)^b^ wks | + | 95% | Premature stop of inclusions | 54/82 (65.9) | 53/89 (59.6) | P=0.38 | 1.1*  (0.9-1.4) | -6.4 %  (-2.1 to 8.1) | High | High |
| **Bradycardia** | | | | | | | | | | | | | | | | |
| HR decrease, mean (+/-SD) | Morphine + atropine + suxamethonium (n=10)  vs no medication (n=10) | Oei | 2002 | J Paediatr Child health | Open RCT | Monocentric  Preterm and term  Intervention:  29 (25-39)^a^ wks  Control:  30.5 (25-40)^a^ wks | + |  | No | -28 (41) bpm | -62 (46) bpm | P=0.104 | - | +34 bpm*  (-7 to 75) | Moderate | High |
| HR<100/min, n/N (%) | Fentanyl + mivacurium + atropine (n=21)  vs fentanyl + atropine (n=20) | Roberts | 2006 | Pediatrics | Open RCT | Bi-centric  Preterm and term  Intervention: 29.7 (24-38)^a^ wks  Control:  30.2 (24-42)^a^ wks | + | - | No | 3/21 (14.3) | 1/20 (5) | P=0.61 | 2.9*  (0.3 -25) | +9.3 %*  (-8.5 to 27) | Moderate | High |
| HR decrease | Morphine + atropine + suxamethonium (n=30)  vs propofol (n=33) | Ghanta | 2007 | Pediatrics | Open RCT | Monocentric  Preterm and term  Morphine+sux: 28 (25-31)^b^ wks  Propofol:  27 (25-30)^b^ wks | + | - | No | Data not available | Data not available | NS  P not provided | - | - | Moderate | Moderate |
| HR decrease, mean (+/-SD) | Fentanyl + suxamethonium + atropine (n=15)  vs atropine + remifentanil (n=15) | Choong | 2010 | Arch Dis Child Fetal Neonatal Ed | Double-blind RCT | Monocentric  Preterm and term  Fentanyl+sux: 27.1 (25.6-28.7)^b^ wks  Remifentanil:  28 (25-30)^b^ wks | + |  | No | -52 (29) bpm | -52 (32) bpm | P=0.98 | - | 0 bpm*  (-23 to 23) | High | High |
| Bradycardia (No definition), n/N (%) | Fentanyl + rocuronium + atropine (n=20) vs fentanyl + atropine (n=24) | Feltman | 2011 | J Perinatol | Open RCT | Monocentric  Preterm<36 wks  Several attempts for one patient taken into account for analysis | + | - | No | 1/57 (1.8) | 2/24 (8.3) | P=0.21* | 0.2*  (0.02 – 2.2) | -6.6 %*  (-18 to 5) | Very Low | Low |
| HR<100/min  > 60 s, n/N (%) | Sufentanil + atracurium + atropine (n=82)  vs propofol + atropine (n=89) | Durrmeyer | 2018 | JAMA | Double-blind RCT | Multicentric  Preterm and term  Sufentanil+atrac: 29 (26-32)^b^ wks  Propofol:  30 (28-34)^b^ wks | + | - | Premature stop of inclusions | 6/80 (7.5) | 1/80 (1.2) | P=0.12* | 1.1*  (0.7 – 49) | +6.3%*  (-0.01 to 13) | High | High |
| **Hypotension** | | | | | | | | | | | | | | | | |
| Change in maximal systolic BP, mean (+/-SD) | Fentanyl + mivacurium + atropine (n=21)  vs fentanyl + atropine (n=20) | Roberts | 2006 | Pediatrics | Open RCT | Bi-centric  Preterm and term  Intervention: 29.7 (24-38)^a^  wks  Control:  30.2 (24-42)^a^ wks | + | - | No | +5.4 (10.2)  (n=17) | +10.1 (12.1)  (n=13) | P=0.27 | - | - | Low | Low |
| Lowest MBP during intubation | Morphine + atropine + suxamethonium (n=30)  vs propofol (n=33) | Ghanta | 2007 | Pediatrics | Open RCT | Monocentric  Preterm and term  Morphine+sux: 28 (25-31)^b^ wks  Propofol:  27 (25-30)^b^ wks | + | - | No | Data not available | Data not available | NS  P not provided | - | - | Moderate | Moderate |
| MBP changes, mean (+/-SD) | Fentanyl + suxamethonium + atropine (n=15)  vs atropine + remifentanil (n=15) | Choong | 2010 | Arch Dis Child Fetal Neonatal Ed | Double-blind RCT | Monocentric  Preterm and term  Fentanyl+sux: 27.1 (25.6-28.7)^b^ wks  Remifentanil:  28 (25-30)^b^ wks | + | 95% | No | +4.3 (7.5) mm Hg | +4.3 (15.9) mm Hg | P=0.98 | - | 0 mmHg*  (-9 to 9) | High | High |
| MBP changes during intubation, mean (+/-SD) | Glycopyrrolate + thiopental + suxamethonium + remifentanil (n=17) vs atropine + morphine (n=17) | Norman | 2011 | J Pediatr | Double-blind RCT | Monocentric  Preterm  Intervention:  27 (25.6-28.5)^b^ wks  Morphine:  26.6 (25.1-28.7)^b^ wks | + | 95% | No | +2 (22) % | +21 (23) % | **P=0.007** | - | -19 %*  (-35 to -3.3) | High | Moderate |
| MBP change 3h after intubation, mean (+/-SD) | Glycopyrrolate + thiopental + suxamethonium + remifentanil (n=17) vs atropine + morphine (n=17) | Norman | 2011 | J Pediatr | Double-blind RCT | Monocentric  Preterm  Intervention: 27 (25.6-28.5)^b^ wks  Morphine: 26.6 (25.1-28.7)^b^ wks | + | 95% | No | -0.62 (5.92) mm Hg | -6.44 (8.49) mm Hg | **P=0.033** | - | +5.8 mm Hg*  (0.7 to 11) | High | Moderate |
| Hypotension (No definition), n/N (%) | Sufentanil + atracurium + atropine (n=82)  vs propofol + atropine (n=89) | Durrmeyer | 2018 | JAMA | Double-blind RCT | Multicentric  Preterm and term  Sufentanil+atrac: 29 (26-32)^b^ wks  Propofol:  30 (28-34)^b^ wks  No inclusion of infants with MBP<GA or CRT>3 s | + | 95% | Premature stop of inclusions | 1/80 (1.3) | 11/83 (13.3) | **P=0.005*** | 0.09*  (0.01 – 0.7) | -12 %*  (-20 to -4) | High | Moderate |
| MBP change 15 min after injection | Sufentanil + atracurium + atropine (n=82)  vs propofol + atropine (n=89) | Durrmeyer | 2018 | JAMA | Double-blind RCT | Multicentric  Preterm and term  Sufentanil+atrac: 29 (26-32)^b^ wks  Propofol: 30 (28-34)^b^ wks  No inclusion of infants with MBP<GA or CRT>3 s | + | 95% | Premature stop of inclusions | 0.2 (12.7) mm Hg | -6.8 (12.7) mm Hg | **P<0.001** | - | +7.0 mm Hg  (3.0 to 11.1) | High | High |
| **Pain and comfort: impossible to assess clinically due the paralyzing effect of muscle blockers** | | | | | | | | | | | | | | | | |
| **Intubation conditions** | | | | | | | | | | | | | | | | |
| « Excellent » Intubation conditions  (Likert scale) according to operator, n/N (%) | Fentanyl + suxamethonium + atropine (n=15)  vs atropine + remifentanil (n=15) | Choong | 2010 | Arch Dis Child Fetal Neonatal Ed | Double-blind RCT | Monocentric  Preterm and term  Fentanyl+sux: 27.1 (25.6-28.7)^b^ wks  Remifentanil:  28 (25-30)^b^ wks | + | 95% | No | 8/15 (53.3) | 1/15 (6.7) | **P=0.009** | 8*  (1.1 - 56) | +47 %*  (18 to 75) | Moderate | High |
| Intubation conditions (Likert scale) according to assistant nurse, % | Fentanyl + suxamethonium + atropine (n=15)  vs atropine + remifentanil (n=15) | Choong | 2010 | Arch Dis Child Fetal Neonatal Ed | Double-blind RCT | Monocentric  Preterm and term  Fentanyl+sux: 27.1 (25.6-28.7)^b^ wks  Remifentanil:  28 (25-30)^b^ wks | + | - | No | % Not available | % Not available | P=0.08 | - | - | Moderate | High |
| Intubation score (Viby-Mogensen), Median [IQR] | Glycopyrrolate + thiopental + suxamethonium + remifentanil (n=17) vs atropine + morphine (n=17) | Norman | 2011 | J Pediatr | Double-blind RCT | Monocentric  Preterm  Intervention:  27 (25.6-28.5)^b^ wks  Morphine:  26.6 (25.1-28.7)^b^ wks | + | - | No | 5 (5-6) | 12 (10-13.5) | **P<0.001** | - | - | High | High |
| Good Intubation conditions (Viby-Mogensen), n/N (%) | Glycopyrrolate + thiopental + suxamethonium + remifentanil (n=17) vs atropine + morphine (n=17) | Norman | 2011 | J Pediatr | Double-blind RCT | Monocentric  Preterm  Intervention:  27 (25.6-28.5)^b^ wks  Morphine:  26.6 (25.1-28.7)^b^ wks | + | 95% | No | 16/17 (94.1) | 1/17 (5.9) | **P<0.001** | 16*  (2.4 – 108) | +88 %*  (72 to 104) | High | High |
| « Excellent » quality of sedation (standardized scale), n/N (%) | Sufentanil + atracurium + atropine (n=82)  vs propofol + atropine (n=89) | Durrmeyer | 2018 | JAMA | Double-blind RCT | Multicentric  Preterm and term  Sufentanil+atrac: 29 (26-32)^b^ wks  Propofol:  30 (28-34)^b^ wks | + | 95% | Premature stop of inclusions | 75/81 (92.6) | 45/87 (51.7) | **P<0.001** | 1.8*  (1.4 – 2.2) | +41 %*  (29 to 53) | High | High |
| * Test performed by the working group  ^a^Mean (range)  ^b^Median (Interquartile range)  Abbreviations: CI, confidence interval; NS, not significant; RCT, randomized controlled trial; wks, weeks of gestation; SpO_2_, oxygen saturation; HR, heart rate; BP, blood pressure; MBP, mean blood pressure; CRT, capillary refill time; GA, gestational age. | | | | | | | | | | | | | | | | |

| **Supplementary Table 3: Randomized trials comparing premedication with an opioid alone versus another regimen for tracheal intubation in neonates** | | | | | | | | | | | | | | | | |  |
| --- | --- | --- | --- | --- | --- | --- | --- | --- | --- | --- | --- | --- | --- | --- | --- | --- | --- |
| **General** | | | | | **Quality** | | | | | **Patient** | | **Effect** | | | **Synthesis** | |  |
| **Outcome** | **Intervention** | **Author** | **Year** | **Journal** | **Type** | **Consistency (I²)** | **Direct effect** | **Precision of CI** | **Other**  **bias?** | **Studied group** | **Control group** | **P-value** | **Relative risk** | **Absolute difference** | **Quality** | **Importance** |  |
| **Number of attempts for intubation** | | | | | | | | | | | | | | | | | |
| N median [IQR] | Morphine (n=17)  vs placebo (n=17) | Lemyre | 2004 | BMC Pediatr | Double-blind RCT | Monocentric  Preterm and term  Morphine:  28 (26-33)^a^ wks  Placebo:  27 (26-30)^a^ wks | + | - | No | 2 (1-3,5) | 1 (1-2,5) | P=0.34 | - | - | High | High |  |
| Success rate at 1^st^ attempt, n/N (%) | Atropine + remifentanil (n=15)  vs fentanyl + suxamethonium + atropine (n=15) | Choong | 2010 | Arch Dis Child Fetal Neonatal Ed | Double-blind RCT | Monocentric  Preterm and term  Fentanyl+sux: 27.1 (25.6-28.7)^a^ wks  Remifentanil: 28 (25-30)^a^ wks | + | 95% | No | 9/15 (60) | 6/15 (40) | P=0.47 | 1.5*  (0.7-3.2) | +20%*  (-15 to 55) | High | High |  |
| N mean (SD) | Atropine + remifentanil (n=15)  vs fentanyl + suxamethonium + atropine (n=15) | Choong | 2010 | Arch Dis Child Fetal Neonatal Ed | Double-blind RCT | Monocentric  Preterm and term  Fentanyl+sux: 27.1 (25.6-28.7)^a^ wks  Remifentanil: 28 (25-30)^a^ wks | + | 95% | No | 1.7 (0.9) | 1.8 (0.8) | P=0.67 | - | -0.1*  (-0.7 to 0.5) | High | High |  |
| N median [IQR] | Atropine + morphine (n=17)  vs Glycopyrrolate + thiopental + suxamethonium + remifentanil (n=17) | Norman | 2011 | J Pediatr | Double-blind RCT | Monocentric Preterm  Intervention: 27 (25.6-28.5)^a^ wks  Morphine:  26.6 (25.1-28.7)^a^ wks | + | - | No | 1 (1-2) | 1 (1-1.5) | NS  P not provided | - | - | High | Moderate |  |
| Success rate at 1^st^ attempt, n/N (%) | Atropine + remifentanil (n=20)  vs atropine (n=20) | Badiee | 2013 | J Res Pharm Pract | Double-blind RCT | Bi-centric  Preterm  Remifentanil: 30.6 (2.8)^b^ wks  Control:  31.7 (3.5)^b^ wks  Exclusion if ≥ 3 attempts | - | 95% | Inadequate randomization | 17/20 (85) | 16/20 (80) | P=0.67 | 1.1*  (0.8 – 1.4) | +5%*  (-18 to 28) | Very Low | Low |  |
| Success rate at 1^st^ attempt, n/N (%) | Remifentanil + atropine (n=36)  vs morphine + midazolam + atropine (n=35) | Avino | 2014 | J Pediatr | Non inferiority open RCT | Monocentric  Preterm and term  Remifentanil: 32.2 (28-38)^a^ wks  Morphine:  34.3 (30-38)^a^ wks  Exclusion if≥ 2 attempts | - | Non inferiority threshold 15% | No | 27/36 (75) | 24/35 (69) | P=0.55 | 1.1*  (0.8 – 1.5) | +6%*  (-14 to 27) | Very Low | Low |  |
| **Duration of intubation** | | | | | | | | | | | | | | | | | |
| Median [IQR] | Morphine (n=17) vs placebo (n=17) | Lemyre | 2004 | BMC Pediatr | Double-blind RCT | Monocentric  Preterm and term  Morphine:  28 (26-33)^a^ wks  Placebo:  27 (26-30)^a^ wks | + | - | No | 271 s  [57,5-418,5] | 94 s  [62,0-215,5] | P=0.27 | - | - | High | High |  |
| Median [IQR] | Atropine + remifentanil (n=15)  vs fentanyl + suxamethonium + atropine (n=15) | Choong | 2010 | Arch Dis Child Fetal Neonatal Ed | Double-blind RCT | Monocentric  Preterm and term  Fentanyl+sux: 27.1 (25.6-28.7)^a^ wks  Remifentanil: 28 (25-30)^a^ wks | + | - | No | 247 s  [48-349] | 156 s  [46-395] | P=0.88 | - | - | High | High |  |
| Median [IQR] | Atropine + morphine (n=17)  vs Glycopyrrolate + thiopental + suxamethonium + remifentanil (n=17) | Norman | 2011 | J Pediatr | Double-blind RCT | Monocentric  Preterm  Intervention: 27 (25.6-28.5)^a^ wks  Morphine:  26.6 (25.1-28.7)^a^ wks | + | - | No | 97 s  [49-365] | 45 s  [35-154] | **P=0.031** | - | - | High | High |  |
| Mean  (+/- SD) | Atropine + remifentanil (n=20)  vs atropine (n=20) | Badiee | 2013 | J Res Pharm Pract | Double-blind RCT | Bi-centric  Preterm  Remifentanil: 30.6 (2.8)^b^ wks  Control:  31.7 (3.5)^b^ wks  Exclusion if ≥ 3 attempts | - | 95% | Inadequate randomization | 20.8 (6.0) s | 22.8 (7.3) s | P=0.34 | - | -2 s*  (-6.3 to 2.3) | Very Low | Moderate |  |
| Duration of 1^st^ attempt, median [IQR] | Remifentanil + atropine (n=36)  vs morphine + midazolam + atropine (n=35) | Avino | 2014 | J Pediatr | Non inferiority open RCT | Monocentric  Preterm and term  Remifentanil: 32.2 (28-38)^a^ wks  Morphine:  34.3 (30-38)^a^ wks  Exclusion if ≥ 2 attempts | + | Non inferiority threshold 15% | No | 33 (24-45) s | 36 (25-59) s | P=0.36 | - | - | Very Low | Low |  |
| Duration of 2^nd^ attempt, median [IQR] | Remifentanil + atropine (n=36)  vs morphine + midazolam + atropine (n=35) | Avino | 2014 | J Pediatr | Non inferiority open RCT | Monocentric  Preterm and term  Remifentanil: 32.2 (28-38)^a^ wks  Morphine:  34.3 (30-38)^a^ wks  Exclusion if ≥ 2 attempts | + | Non inferiority threshold 15% | No | 45 (35-64) s | 56 (44-68) s | P=0.30 | - | - | Very Low | Low |  |
| **Hypoxia** | | | | | | | | | | | | | | | | | |
| SpO_2_<85%, n/N (%) | Morphine (n=17)  vs placebo (n=17) | Lemyre | 2004 | BMC Pediatr | Double-blind RCT | Monocentric  Preterm and term  Morphine:  28 (26-33)^a^ wks  Placebo:  27 (26-30)^a^ wks | + | 95% | No | 17/17 (100) | 14/17 (82%) | P=0,23* | 1.2*  (0.97 –1.5) | +18 %*  (-0.5 to 36) | High | High |  |
| Delta SpO_2_, mean  (+ /- SD) | Atropine + remifentanil (n=15)  vs fentanyl + suxamethonium + atropine (n=15) | Choong | 2010 | Arch Dis Child Fetal Neonat Ed | Double-blind RCT | Monocentric  Preterm and term  Fentanyl+sux: 27.1 (25.6-28.7)^a^ wks  Remifentanil: 28 (25-30)^a^ wks | + | 95% | No | - 55 (27) % | - 47 (25) % | P=0.42 | - | -8 %*  (-27 to 11) | High | High |  |
| SpO_2_ mean | Atropine + morphine (n=17)  vs Glycopyrrolate + thiopental + suxamethonium + remifentanil (n=17) | Norman | 2011 | J Pediatr | Double-blind RCT | Monocentric  Preterm  Intervention: 27 (25.6-28.5)^a^ wks  Morphine:  26.6 (25.1-28.7)^a^ wks | + | - | No | Data not available | Data not available | NS  P not provided | - | - | High | Moderate |  |
| SpO_2_<70%, n/N (%) | Atropine + remifentanil (n=20)  vs atropine (n=20) | Badiee | 2013 | J Res Pharm Pract | Double-blind RCT | Bi-centric  Preterm  Remifentanil: 30.6 (2.8)^b^ wks  Control:  31.7 (3.5)^b^ wks  Exclusion if ≥ 3 attempts | - | 95% | Inadequate randomization | 4/20 (20) | 1/20 (5) | P=0.17 | 4*  (0.5 - 33) | +15 %*  ( -5 to 35) | Very Low | Moderate |  |
| SpO_2_ values at 1, 3, 5, 10, 15, 30 and 60 min (1^st^ attempt) | Remifentanil + atropine (n=36)  vs morphine + midazolam + atropine (n=35) | Avino | 2014 | J Pediatr | Non inferiority open RCT | Monocentric  Preterm and term  Remifentanil: 32.2 (28-38)^a^ wks  Morphine:  34.3 (30-38)^a^ wks  Exclusion if ≥ 2 attempts | + | Non inferiority threshold 15% | No | Data not available | Data not available | NS  P not provided | - | - | Low | Low |  |
| SpO_2_ values at 5, 30 and 60 min (2^nd^ attempt) | Remifentanil + atropine (n=36)  vs morphine + midazolam + atropine (n=35) | Avino | 2014 | J Pediatr | Non inferiority open RCT | Monocentric  Preterm and term  Remifentanil: 32.2 (28-38)^a^ wks  Morphine:  34.3 (30-38)^a^ wks  Exclusion if ≥ 2 attempts | + | Non inferiority threshold 15% | No | Data not available | Data not available | **P=0.031 (M5)**  **P=0.042 (M30)**  **P=0.07 (M60)** | - | - | Low | Low |  |
| SpO_2_ values at 1, 3, 10 et 15 min (2^nd^ attempt) | Remifentanil + atropine (n=36)  vs morphine + midazolam + atropine (n=35) | Avino | 2014 | J Pediatr | Non inferiority open RCT | Monocentric  Preterm and term  Remifentanil: 32.2 (28-38)^a^ wks  Morphine:  34.3 (30-38)^a^ wks  Exclusion if ≥ 2 attempts | + | Non inferiority threshold 15% | No | Data not available | Data not available | NS  P not provided | - | - | Low | Low |  |
| **Bradycardia** | | | | | | | | | | | | | | | | | |
| HR<90/min, n/N (%) | Morphine (n=17) vs placebo (n=17) | Lemyre | 2004 | BMC Pediatr | Double-blind RCT | Monocentric  Preterm and term  Morphine:  28 (26-33)^a^ wks  Placebo:  27 (26-30)^a^ wks | + | 95% | No | 16/17 (94.1) | 12/17 (70.6) | P=0.18 | 1.3*  (0.96 –1.9) | +24 %*  (-0.8 to 48) | High | High |  |
| HR decrease, mean (+/-SD) | Atropine + remifentanil (n=15)  vs fentanyl + suxamethonium + atropine (n=15) | Choong | 2010 | Arch Dis Child Fetal Neonatal Ed | Double-blind RCT | Monocentric  Preterm and term  Fentanyl+sux: 27.1 (25.6-28.7)^a^ wks  Remifentanil: 28 (25-30)^a^ wks | + | 95% | No | -52 (31,6) bpm | -52 (29) bpm | P=0.98 | - | 0 bpm*  (-23 to 23) | High | High |  |
| Bradycardia (no definition), n/N (%) | Remifentanil + atropine (n=36)  vs morphine + midazolam + atropine (n=35) | Avino | 2014 | J Pediatr | Non inferiority open RCT | Monocentric  Preterm and term  Remifentanil: 32.2 (28-38)^a^ wks  Morphine:  34.3 (30-38)^a^ wks  Exclusion if ≥ 2 attempts | + | Non inferiority threshold 15% | No | 0/36 (0) | 0/35 (0) | NA | - | - | Low | Moderate |  |
| **Hypotension** | | | | | | | | | | | | | | | | | |
| MBP change, mean (+/-SD) | Atropine + remifentanil (n=15)  vs fentanyl + suxamethonium + atropine (n=15) | Choong | 2010 | Arch Dis Child Fetal Neonatal Ed | Double-blind RCT | Monocentric  Preterm and term  Fentanyl+sux: 27.1 (25.6-28.7)^a^ wks  Remifentanil: 28 (25-30)^a^ wks | + | 95% | No | +4.3 (15.9) mm Hg | +4.3 (7.5) mm Hg | P=0,98 | - | 0 mm Hg*  (-9 to 9) | High | High |  |
| MBP 5 min after intubation | Atropine + remifentanil (n=20)  vs atropine (n=20) | Badiee | 2013 | J Res Pharm Pract | Double-blind RCT | Bi-centric  Preterm  Remifentanil: 30.6 (2.8)^b^ wks  Control:  31.7 (3.5)^b^ wks  Exclusion if ≥ 3 attempts | + | - | Inadequate randomization | 43.6 | 43.0 | - | - | - | Very Low | Low |  |
| Hypotension (no definition), n/N (%) | Remifentanil + atropine (n=36)  vs morphine + midazolam + atropine (n=35) | Avino | 2014 | J Pediatr | Non inferiority open RCT | Monocentric  Preterm and term  Remifentanil: 32.2 (28-38)^a^ wks  Morphine:  34.3 (30-38)^a^ wks  Exclusion if ≥ 2 attempts | + | Non inferiority threshold 15% | No | 0/36 (0) | 0/35 (0) | NA | - | - | Low | Moderate |  |
| MBP at 1, 3, 5, 10, 15, 30 and 60 min (1^st^ attempt) | Remifentanil + atropine (n=36)  vs morphine + midazolam + atropine (n=35) | Avino | 2014 | J Pediatr | Non inferiority open RCT | Monocentric  Preterm and term  Remifentanil: 32.2 (28-38)^a^ wks  Morphine:  34.3 (30-38)^a^ wks  Exclusion if ≥ 2 attempts | + | Non inferiority threshold 15% | No | Data not available | Data not available | NS  P not provided | - | - | Low | Low |  |
| **Pain and comfort** | | | | | | | | | | | | | | | | | |
| PIPP score, mean (+/-SD) | Atropine + remifentanil (n=20)  vs atropine (n=20) | Badiee | 2013 | J Res Pharm Pract | Double-blind RCT | Bi-centric  Preterm  Remifentanil: 30.6 (2.8)^b^ wks  Control:  31.7 (3.5)^b^ wks  Exclusion if ≥ 3 attempts | + | 95% | Inadequate randomization | 7.5 (1.4) | 15.1 (1.6) | **P<0.001** | - | -7.6*  (-8.6 to -6.6) | Very Low | High |  |
| DAN scale at 10 min after 1^st^ attempt, median [IQR] | Remifentanil + atropine (n=36)  vs morphine + midazolam + atropine (n=35) | Avino | 2014 | J Pediatr | Non inferiority open RCT | Monocentric  Preterm and term  Remifentanil: 32.2 (28-38)^a^ wks  Morphine:  34.3 (30-38)^a^ wks  Exclusion if ≥ 2 attempts | + | HR | No | 2 [0-3] | 0.5 [0-2] | **0.026** | - | - | Low | High |  |
| DAN scale at 10 min after 2^nd^ attempt, median [IQR] | Remifentanil + atropine (n=36)  vs morphine + midazolam + atropine (n=35) | Avino | 2014 | J Pediatr | Non inferiority open RCT | Monocentric  Preterm and term  Remifentanil: 32.2 (28-38)^a^ wks  Morphine:  34.3 (30-38)^a^ wks  Exclusion if ≥ 2 attempts | + | Non inferiority threshold 15% | No | 4 [0.8-8.3] | 0 [0-1.3] | **0.042** | - | - | Low | High |  |
| **Intubation conditions** | | | | | | | | | | | | | | | | | |
| Intubation conditions  « Excellent » (Likert scale) according to operator, n/N (%) | Fentanyl + suxamethonium + atropine (n=15)  vs atropine + remifentanil (n=15) | Choong | 2010 | Arch Dis Child Fetal Neonatal Ed | Double-blind RCT | Monocentric  Preterm and term  Fentanyl+sux: 27.1 (25.6-28.7)^a^ wks  Remifentanil: 28 (25-30)^a^ wks | + | 95% | No | 1/15 (6.7) | 8/15 (53.3) | **P=0.009** | 0.12*  (0.02-0.88) | -47%*  (-75 to -18) | High | High |  |
| Chest rigidity, n/N (%) | Atropine + remifentanil (n=15)  vs fentanyl + suxamethonium + atropine (n=15) | Choong | 2010 | Arch Dis Child Fetal Neonatal Ed | Double-blind RCT | Monocentric  Preterm and term  Fentanyl+sux: 27.1 (25.6-28.7)^a^ wks  Remifentanil: 28 (25-30)^a^ wks | + | 95% | No | 2/15 (13.3) | 0/15 (0) | P=0.48 | - | +13 %*  (-4 to 31) | High | High |  |
| Chest rigidity, n/N (%) | Atropine + remifentanil (n=20)  vs atropine (n=20) | Badiee | 2013 | J Res Pharm Pract | Double-blind RCT | Bi-centric  Preterm  Remifentanil: 30.6 (2.8)^b^ wks  Control:  31.7 (3.5)^b^ wks  Exclusion if ≥ 3 attempts | + | 95% | Inadequate randomization | 4/20 (20) | 0/20 (0) | P=0.11* | - | +20 %*  (2.5 to 38) | Very Low | High |  |
| Poor Intubation conditions, n/N (%) | Remifentanil + atropine (n=36)  vs morphine + midazolam + atropine (n=35) | Avino | 2014 | J Pediatr | Non inferiority open RCT | Monocentric  Preterm and term  Remifentanil: 32.2 (28-38)^a^ wks  Morphine:  34.3 (30-38)^a^ wks  Exclusion if ≥ 2 attempts | + | Non inferiority threshold 15% | No | 11/36 (30.6) | 11/35 (31.4) | P=0.47 | 0.97*  (0.5 – 1.9) | -0.9 %*  (-22 to 21) | Low | High |  |
| * Test performed by the working group  ^a^Median (IQR)  ^b^Mean (SD)  Abbreviations: CI, confidence interval; NS, not significant; RCT, randomized controlled trial; SpO_2_, oxygen saturation; HR, heart rate; BP, blood pressure; MBP, mean blood pressure; PIPP, premature infant pain profile; DAN, douleur aiguë du nouveau-né. | | | | | | | | | | | | | | | | |  |

| **Supplementary Table 4: Randomized trials comparing premedication with midazolam alone or in association versus another regimen for tracheal intubation in neonates** | | | | | | | | | | | | | | | | |  |
| --- | --- | --- | --- | --- | --- | --- | --- | --- | --- | --- | --- | --- | --- | --- | --- | --- | --- |
| **General** | | | | | **Quality** | | | | | **Patient** | | **Effect** | | | **Synthesis** | |  |
| **Outcome** | **Intervention** | **Author** | **Year** | **Journal** | **Type** | **Consistency (I²)** | **Direct effect** | **Precision of CI** | **Other**  **bias?** | **Studied group** | **Control group** | **P-value** | **Relative risk** | **Absolute difference** | **Quality** | **Importance** |  |
| **Number of attempts for intubation** | | | | | | | | | | | | | | | | | |
| Success rate at 1^st^ attempt, n/N (%) | Remifentanil + midazolam (n=10)  vs remifentanil + propofol (n=10) | Penido | 2011 | J Perinatol | Double-blind RCT | Monocentric  Preterm 28-34 wks  Midazolam:  32 (1.6)^a^ wks  Propofol:  31.5 (1.5)^a^ wks  Single operator | + | 95% | No | 7/10 (70) | 8/10 (80) | P=1.00 | 0.9*  (0.5 – 1.5) | -10 %*  (-48 to 28) | Moderate | High |  |
| Success rate at 1^st^ attempt, n/N (%) | Morphine + midazolam + atropine (n=35)  vs remifentanil + atropine (n=36) | Avino | 2014 | J Pediatr | Open RCT non-inferiority | Monocentric  Preterm and term  Remifentanil: 32.2 (28-38)^b^ wks  Morphine:  34.3 (30-38)^b^ wks  Exclusion if failure after 2 attempts | + | Non-inferiority threshold: 15% | No | 24/35 (69) | 27/36 (75) | P=0.55 | 0.9*  (0.7 – 1.2) | -6 %*  (-27 to 14) | Very Low | Low |  |
| Success rate at 1^st^ attempt, % | Atropine + midazolam (n=40)  vs atropine + placebo (n=40) | Badiee | 2021 | J Res Med Sci | Double-blind RCT | Monocentric  Preterm  Midazolam: 31.8 (2.6)^a^ wks  Control:  29.8 (0.3)^a^ wks  Exclusion if ≥ 2 attempts | - | 95% | Ambiguous randomization | n not available  92.5 | n not available  66.7 | **P=0.004** | - | - | Very Low | Low |  |
| **Duration of intubation** | | | | | | | | | | | | | | | | | |
| Duration of 1^st^ intubation attempt, median [IQR] | Morphine + midazolam + atropine (n=35)  vs remifentanil + atropine (n=36) | Avino | 2014 | J Pediatr | Open RCT non-inferiority | Monocentric  Preterm and term  Remifentanil: 32.2 (28-38)^b^ wks  Morphine:  34.3 (30-38)^b^ wks  Exclusion if failure after 2 attempts | + | Non-inferiority threshold: 15% | No | 36 s  [25-59] | 33 s  [24-45] | P=0.36 | - | - | Very Low | Low |  |
| Duration of 2^nd^ intubation attempt, median [IQR] | Morphine + midazolam + atropine (n=35)  vs remifentanil + atropine (n=36) | Avino | 2014 | J Pediatr | Open RCT non-inferiority | Monocentric  Preterm and term  Remifentanil: 32.2 (28-38)^b^ wks  Morphine:  34.3 (30-38)^b^ wks  Exclusion if failure after 2 attempts | + | Non-inferiority threshold: 15% | No | 56 s  [44-68] | 45 s  [35-64] | P=0.30 | - | - | Very Low | Low |  |
| Mean  (+/- SD) | Atropine + midazolam (n=40)  vs atropine + placebo (n=40) | Badiee | 2021 | J Res Med Sci | Double-blind RCT | Monocentric  Preterm  Midazolam: 31.8 (2.6)^a^ wks  Control:  29.8 (0.3)^a^ wks  Exclusion if ≥ 2 attempts | + | 95% | Ambiguous randomization | 18.8 (4.8) s | 23.5 (6.7) s | **P=0.001** | - | -4.7 s*  (-7.3 to -2.1) | Very Low | Moderate |  |
| **Hypoxia** | | | | | | | | | | | | | | | | | |
| SpO_2_ values at 1, 3, 5, 10, 15, 30 and 60 min (1^st^ attempt) | Morphine + midazolam + atropine (n=35)  vs remifentanil + atropine (n=36) | Avino | 2014 | J Pediatr | Open RCT non-inferiority | Monocentric  Preterm and term  Remifentanil: 32.2 (28-38)^b^ wks  Morphine:  34.3 (30-38)^b^ wks  Exclusion if failure after 2 attempts | + | Non-inferiority threshold: 15% | No | Data not available | Data not available | NS  P not available | - | - | Low | Low |  |
| SpO_2_ values at 5, 30 and 60 min (2^nd^ attempt) | Morphine + midazolam + atropine (n=35)  vs remifentanil + atropine (n=36) | Avino | 2014 | J Pediatr | Open RCT non-inferiority | Monocentric  Preterm and term  Remifentanil: 32.2 (28-38)^b^ wks  Morphine:  34.3 (30-38)^b^ wks  Exclusion if failure after 2 attempts | + | Non-inferiority threshold: 15% | No | Data not available | Data not available | **P=0.031 (M5)**  **P=0.042 (M30)**  **P=0.007 (M60)** | - | - | Low | Low |  |
| SpO_2_ values at 1, 3, 10 and 15 min (2^nd^ attempt) | Morphine + midazolam + atropine (n=35)  vs remifentanil + atropine (n=36) | Avino | 2014 | J Pediatr | Open RCT non-inferiority | Monocentric  Preterm and term  Remifentanil: 32.2 (28-38)^b^ wks  Morphine:  34.3 (30-38)^b^ wks  Exclusion if failure after 2 attempts | + | Non-inferiority threshold: 15% | No | Data not available | Data not available | NS  P not available | - | - | Low | Low |  |
| SpO_2_<70%, n/N (%) | Atropine + midazolam (n=40)  vs atropine + placebo (n=40) | Badiee | 2021 | J Res Med Sci | Double-blind RCT | Monocentric  Preterm  Midazolam: 31.8 (2.6)^a^ wks  Control:  29.8 (0.3)^a^ wks  Exclusion if ≥ 2 attempts | + | 95% | Ambiguous randomization | 2/40 (5%) | 22/40 (55%) | **P=10^-4^** | 0.09*  (0.02 – 0.36) | -50 %*  (-67 to -33) | Very Low | Moderate |  |
| **Bradycardia** | | | | | | | | | | | | | | | | | |
| HR<100/min, n/N (%) | Remifentanil + midazolam (n=10)  vs remifentanil + propofol (n=10) | Penido | 2011 | J Perinatol | Double-blind RCT | Monocentric  Preterm 28-34 wks  Midazolam:  32 (1.6)^a^ wks  Propofol:  31.5 (1.5)^a^ wks  Single operator | + | 95% | No | 0/10 (0) | 1/10 (10) | P=1.00* | - | -10 %*  (-29 to 8.6) | Moderate | Moderate |  |
| Bradycardia (no definition), n/N (%) | Morphine + midazolam + atropine (n=35)  vs remifentanil + atropine (n=36) | Avino | 2014 | J Pediatr | Open RCT non-inferiority | Monocentric  Preterm and term  Remifentanil: 32.2 (28-38)^b^ wks  Morphine:  34.3 (30-38)^b^ wks  Exclusion if failure after 2 attempts | + | Non-inferiority threshold: 15% | No | 0/35 (0) | 0/36 (0) | **-** | - | - | Low | Low |  |
| **Hypotension** | | | | | | | | | | | | | | | | | |
| MBP < 25 mm Hg, n/N (%) | Remifentanil + midazolam (n=10)  vs remifentanil + propofol (n=10) | Penido | 2011 | J Perinatol | Double-blind RCT | Monocentric  Preterm 28-34 wks  Midazolam:  32 (1.6)^a^ wks  Propofol:  31.5 (1.5)^a^ wks  Single operator | + | 95% | No | 2/10 (20) | 2/10 (20) | P=1.00* | 1* (0.2 – 5.8) | 0 %*  (-35 to 35) | Moderate | Moderate |  |
| Hypotension (no definition), n/N (%) | Morphine + midazolam + atropine (n=35)  vs remifentanil + atropine (n=36) | Avino | 2014 | J Pediatr | Open RCT non-inferiority | Monocentric  Preterm and term  Remifentanil: 32.2 (28-38)^b^ wks  Morphine:  34.3 (30-38)^b^ wks  Exclusion if failure after 2 attempts | + | Non-inferiority threshold: 15% | No | 0/35 (0) | 0/36 (0) | **-** | - | - | Low | Low |  |
| MBP 5 min after intubation | Atropine + midazolam (n=40)  vs atropine + placebo (n=40) | Badiee | 2021 | J Res Med Sci | Double-blind RCT | Monocentric  Preterm  Midazolam: 31.8 (2.6)^a^ wks  Control:  29.8 (0.3)^a^ wks  Exclusion if ≥ 2 attempts | + | - | Ambiguous randomization | 43.6 | 43.0 | - | - | - | Very Low | Low |  |
| **Pain and comfort** | | | | | | | | | | | | | | | | | |
| Pain (NIPS scale) | Remifentanil + midazolam (n=10)  vs remifentanil + propofol (n=10) | Penido | 2011 | J Perinatol | Double-blind RCT | Monocentric  Preterm 28-34 wks  Midazolam:  32 (1.6)^a^ wks  Propofol:  31.5 (1.5)^a^ wks  Single operator | + | - | No | Data not available | Data not available | P=1.00 | - | - | Moderate | Moderate |  |
| Pain (COMFORT scale) | Remifentanil + midazolam (n=10)  vs remifentanil + propofol (n=10) | Penido | 2011 | J Perinatol | Double-blind RCT | Monocentric  Preterm 28-34 wks  Midazolam:  32 (1.6)^a^ wks  Propofol:  31.5 (1.5)^a^ wks  Single operator | + | - | No | Data not available | Data not available | P=0.33 | - | - | Moderate | Moderate |  |
| DAN scale 10 min after 1^st^ attempt, median [IQR] | Morphine + midazolam + atropine (n=35)  vs remifentanil + atropine (n=36) | Avino | 2014 | J Pediatr | Open RCT non-inferiority | Monocentric  Preterm and term  Remifentanil: 32.2 (28-38)^b^ wks  Morphine:  34.3 (30-38)^b^ wks  Exclusion if failure after 2 attempts | + | Non-inferiority threshold: 15% | No | 0.5 [0-2] | 2 [0-3] | **P=0.026** | - | - | Low | High |  |
| DAN scale 10 min after 2^nd^ attempt, median [IQR] | Morphine + midazolam + atropine (n=35)  vs remifentanil + atropine (n=36) | Avino | 2014 | J Pediatr | Open RCT non-inferiority | Monocentric  Preterm and term  Remifentanil: 32.2 (28-38)^b^ wks  Morphine:  34.3 (30-38)^b^ wks  Exclusion if failure after 2 attempts | + | Non-inferiority threshold: 15% | No | 0 [0-1.3] | 4 [0.8-8.3] | **P=0.042** | - | - | Low | High |  |
| PIPP score after intubation, mean (+/- SD) | Atropine + midazolam (n=40)  vs atropine + placebo (n=40) | Badiee | 2021 | J Res Med Sci | Double-blind RCT | Monocentric  Preterm  Midazolam: 31.8 (2.6)^a^ wks  Control:  29.8 (0.3)^a^ wks  Exclusion if ≥ 2 attempts | + | 95% | Ambiguous randomization | 5.2 (2.06) | 12.9 (2.9) | **P=10^-4^** | - | -7.7*  (-8.8 to -6.6) | Very Low | Moderate |  |
| FANS score after intubation, mean (+/- SD) | Atropine + midazolam (n=40)  vs atropine + placebo (n=40) | Badiee | 2021 | J Res Med Sci | Double-blind RCT | Monocentric  Preterm  Midazolam: 31.8 (2.6)^a^ wks  Control:  29.8 (0.3)^a^ wks  Exclusion if ≥ 2 attempts | + | 95% | Ambiguous randomization | 0.68 (1.7) | 6.1 (1.5) | **P=10^-4^** | - | -5.4*  (-6.1 to -4.7) | Very Low | Moderate |  |
| **Intubation conditions** | | | | | | | | | | | | | | | | | |
| Excellent conditions (Viby Mogensen Score), n/N (%) | Remifentanil + midazolam (n=10)  vs remifentanil + propofol (n=10) | Penido | 2011 | J Perinatol | Double-blind RCT | Monocentric  Preterm 28-34 wks  Midazolam:  32 (1.6)^a^ wks  Propofol:  31.5 (1.5)^a^ wks  Single operator | + | 95% | No | 3/10 (30) | 4/10 (40) | P=0.73 | 0.75*  (0.2 – 2.5) | -10 %*  (-52 to 32) | Moderate | Moderate |  |
| Poor intubation conditions, n/N (%) | Morphine + midazolam + atropine (n=35)  vs remifentanil + atropine (n=36) | Avino | 2014 | J Pediatr | Open RCT non-inferiority | Monocentric  Preterm and term  Remifentanil: 32.2 (28-38)^b^ wks  Morphine:  34.3 (30-38)^b^ wks  Exclusion if failure after 2 attempts | + | Non-inferiority threshold: 15% | No | 11/35 (31.4) | 11/36 (30.6) | P=0.47 | 1*  (0.5 – 2.1) | +0.9 %*  (-21 to 22) | Low | High |  |
| Excellent conditions (Hans-Cooper), n/N (%) | Atropine + midazolam (n=40)  vs atropine + placebo (n=40) | Badiee | 2021 | J Res Med Sci | Double-blind RCT | Monocentric  Preterm  Midazolam: 31.8 (2.6)^a^ wks  Control:  29.8 (0.3)^a^ wks  Exclusion if ≥ 2 attempts | + | 95% | Ambiguous randomization | 33/40 (82.5) | 0/40 (0) | **P=10^-4^** | - | +82.5 %*  (71 to 94) | Very Low | Low |  |
| * Test performed by the working group  ^a^Mean (SD)  ^b^Median (IQR)  Abbreviations: CI, confidence interval; NS, not significant; RCT, randomized controlled trial; SpO_2_, oxygen saturation; HR, heart rate; BP, blood pressure; MBP, mean blood pressure; NIPS, neonatal infant pain scale ; DAN, douleur aiguë du nouveau-né ; PIPP, premature infant pain profile ; FANS, faceless acute neonatal pain score. | | | | | | | | | | | | | | | | |  |

| **Supplementary Table 5: Randomized trials comparing premedication with propofol versus another regimen for tracheal intubation in neonates** | | | | | | | | | | | | | | | | |
| --- | --- | --- | --- | --- | --- | --- | --- | --- | --- | --- | --- | --- | --- | --- | --- | --- |
| **General** | | | | | **Quality** | | | | | **Patient** | | **Effect** | | | **Synthesis** | |
| **Outcome** | **Intervention** | **Author** | **Year** | **Journal** | **Type** | **Consistency (I²)** | **Direct effect** | **Precision of CI** | **Other**  **bias?** | **Studied group** | **Control group** | **P-value** | **Relative risk** | **Absolute difference** | **Quality** | **Importance** |
| **Number of attempts for intubation** | | | | | | | | | | | | | | | | |
| N median [IQR] | Propofol (n=33) vs morphine + atropine + suxamethonium (n=30) | Ghanta | 2007 | Pediatrics | Open RCT | Monocentric  Preterm and term  Morphine+sux: 28 (25-31)^a^ wks  Propofol:  27 (25-30)^a^ wks | + | - | No | 1 [1-2] | 2 [1-3] | P=0.08 | - | - | Moderate | High |
| Success rate at 1^st^ attempt, n/N (%) | Remifentanil + propofol (n=10)  vs remifentanil + midazolam (n=10) | Penido | 2011 | J Perinatol | Double-blind RCT | Monocentric  Preterm 28-34 wks  Midazolam:  32 (1.6)^b^ wks  Propofol:  31.5 (1.5)^b^ wks  Single operator | + | 95% | No | 8/10 (80) | 7/10 (70) | P=1.00 | 1.1*  (0.7 – 1.9) | +10%*  (-28 to 48) | Moderate | High |
| Success rate at 1^st^ attempt, n/N (%) | Propofol + atropine (n=89)  vs sufentanil + atracurium + atropine (n=82) | Durrmeyer | 2018 | JAMA | Double-blind RCT | Multicentric  Preterm and term  Sufentanil+atrac: 29 (26-32)^a^ wks  Propofol:  30 (28-34)^a^ wks | + | 95% | Premature stop of inclusions | 41/87 (47.1) | 47/81 (58.0) | P=0.37 | 0.8*  (0.6 – 1.1) | -10.9%*  (-26 to 4.1) | High | High |
| N median [IQR] | Propofol + atropine (n=89)  vs sufentanil + atracurium + atropine (n=82) | Durrmeyer | 2018 | JAMA | Double-blind RCT | Multicentric  Preterm and term  Sufentanil+atrac: 29 (26-32)^a^ wks  Propofol:  30 (28-34)^a^ wks | + | 95% | Premature stop of inclusions | 1 [1-2] | 2 [1-2] | P=0.1 | - | 0 | High | High |
| **Duration of intubation** | | | | | | | | | | | | | | | | |
| Median [IQR] | Propofol (n=33) vs morphine + atropine + suxamethonium (n=30) | Ghanta | 2007 | Pediatrics | Open RCT | Monocentric  Preterm and term  Morphine+sux: 28 (25-31)^a^ wks  Propofol:  27 (25-30)^a^ wks | + | - | No | 120 s  [60-180] | 260 s  [60-435] | **P=0.007** | - | - | Moderate | High |
| Median [IQR] | Propofol + atropine (n=89)  vs sufentanil + atracurium + atropine (n=82) | Durrmeyer | 2018 | JAMA | Double-blind RCT | Multicentric  Preterm and term  Sufentanil+atrac: 29 (26-32)^a^ wks  Propofol:  30 (28-34)^a^ wks | + | 95% | Premature stop of inclusions | 6.0 min  [2.8- 9.1] | 3.5 min  [1.3- 6.0] | **P=0.003** | - | +1.7 min  (0.6 to 3.0) | High | High |
| **Hypoxia** | | | | | | | | | | | | | | | | |
| SpO_2_ median [IQR] | Propofol (n=33) vs morphine + atropine + suxamethonium (n=30) | Ghanta | 2007 | Pediatrics | Open RCT | Monocentric  Preterm and term  Morphine+sux: 28 (25-31)^a^ wks  Propofol:  27 (25-30)^a^ wks | + | - | No | 80 [67-88] | 60 [43-82] | **P=0.019** | - | - | Moderate | High |
| SpO_2_<80%  > 60 s, n/N (%) | Propofol + atropine (n=89)  vs sufentanil + atracurium + atropine (n=82) | Durrmeyer | 2018 | JAMA | Double-blind RCT | Multicentric  Preterm and term  Sufentanil+atrac: 29 (26-32)^a^ wks  Propofol:  30 (28-34)^a^ wks | + | 95% | Premature stop of inclusions | 53/89 (59.6) | 54/82 (65.9) | P=0.38 | 0.9*  (0.7 – 1.1) | -6.3 %  (-21 to 8.2) | High | High |
| **Bradycardia** | | | | | | | | | | | | | | | | |
| HR decrease | Propofol (n=33) vs morphine + atropine + suxamethonium (n=30) | Ghanta | 2007 | Pediatrics | Open RCT | Monocentric  Preterm and term  Morphine+sux: 28 (25-31)^a^ wks  Propofol:  27 (25-30)^a^ wks | + | - | No | Data not available | Data not available | NS  P not provided | - | - | Moderate | Moderate |
| HR<100/min, n/N (%) | Remifentanil + propofol (n=10)  vs remifentanil + midazolam (n=10) | Penido | 2011 | J Perinatol | Double-blind RCT | Monocentric  Preterm 28-34 wks  Midazolam:  32 (1.6)^b^ wks  Propofol:  31.5 (1.5)^b^ wks  Single operator | + | 95% | No | 1/10 (10) | 0/10 (0) | P=1.00* | - | +10 %*  (-8.6 to 29) | Moderate | Moderate |
| HR<100/min  > 60 s, n/N (%) | Propofol + atropine (n=89)  vs sufentanil + atracurium + atropine (n=82) | Durrmeyer | 2018 | JAMA | Double-blind RCT | Multicentric  Preterm and term  Sufentanil+atrac: 29 (26-32)^a^ wks  Propofol:  30 (28-34)^a^ wks | + | 95% | Premature stop of inclusions | 1/80 (1.2) | 6/80 (7.5) | P=0.12* | 0.17*  (0.02 – 1.35) | -6.3 %*  (-13 to 0.01) | High | High |
| **Hypotension** | | | | | | | | | | | | | | | | |
| MBP minimum during intubation | Propofol (n=33) vs morphine + atropine + suxamethonium (n=30) | Ghanta | 2007 | Pediatrics | Open RCT | Monocentric  Preterm and term  Morphine+sux: 28 (25-31)^a^ wks  Propofol:  27 (25-30)^a^ wks | + | - | No | Data not available | Data not available | NS  P not provided | - | - | Moderate | Moderate |
| MBP < 25 mm Hg, n/N (%) | Remifentanil + propofol (n=10)  vs remifentanil + midazolam (n=10) | Penido | 2011 | J Perinatol | Double-blind RCT | Monocentric  Preterm 28-34 wks  Midazolam:  32 (1.6)^b^ wks  Propofol:  31.5 (1.5)^b^ wks  Single operator | + | 95% | No | 2/10 (20) | 2/10 (20) | P=1.00* | 1*  (0.2 – 5.8) | 0 %*  (-35 to 35) | Moderate | Moderate |
| Hypotension (no definition), n/N (%) | Propofol + atropine (n=89)  vs sufentanil + atracurium + atropine (n=82) | Durrmeyer | 2018 | JAMA | Double-blind RCT | Multicentric  Preterm and term  Sufentanil+atrac: 29 (26-32)^a^ wks  Propofol:  30 (28-34)^a^ wks  No inclusion of infants with MBP<GA or CRT>3 s | + | 95% | Premature stop of inclusions | 11/83 (13.3) | 1/80 (1.3) | **P=0.005*** | 10.6*  (1.4 – 80) | +12 %*  (4.3 to 20) | High | Moderate |
| MBP change 15 min after injection | Propofol + atropine (n=89)  vs sufentanil + atracurium + atropine (n=82) | Durrmeyer | 2018 | JAMA | Double-blind RCT | Multicentric  Preterm and term  Sufentanil+atrac: 29 (26-32)^a^ wks  Propofol:  30 (28-34)^a^ wks  No inclusion of infants with MBP<GA or CRT>3 s | + | 95% | Premature stop of inclusions | -6.8 (12.7) mm Hg | 0.2 (12.7)  mm Hg | **P<0.001** | - | -7.0 mm Hg  (-3.0 to -11.1) | High | High |
| **Pain and comfort** | | | | | | | | | | | | | | | | |
| Pain (NIPS scale) | Remifentanil + propofol (n=10)  vs remifentanil + midazolam (n=10) | Penido | 2011 | J Perinatol | Double-blind RCT | Monocentric  Preterm 28-34 wks  Midazolam:  32 (1.6)^b^ wks  Propofol:  31.5 (1.5)^b^ wks  Single operator | + | - | No | Data not available | Data not available | P=1.00 | - | - | Moderate | Moderate |
| Pain (COMFORT scale) | Remifentanil + propofol (n=10)  vs remifentanil + midazolam (n=10) | Penido | 2011 | J Perinatol | Double-blind RCT | Monocentric  Preterm 28-34 wks  Midazolam:  32 (1.6)^b^ wks  Propofol:  31.5 (1.5)^b^ wks  Single operator | + | - | No | Data not available | Data not available | P=0.33 | - | - | Moderate | Moderate |
| **Intubation conditions** | | | | | | | | | | | | | | | | |
| Excellent conditions (Viby Mogensen score), n/N (%) | Remifentanil + propofol (n=10)  vs remifentanil + midazolam (n=10) | Penido | 2011 | J Perinatol | Double-blind RCT | Monocentric  Preterm 28-34 wks  Midazolam:  32 (1.6)^b^ wks  Propofol:  31.5 (1.5)^b^ wks  Single operator | + | 95% | No | 4/10 (40) | 3/10 (30) | P=0.73 | 1.3*  (0.4 – 4.5) | +10 %*  (-32 to 52) | Moderate | Moderate |
| “Excellent” quality of sedation (standardized scale), n/N (%) | Propofol + atropine (n=89)  vs sufentanil + atracurium + atropine (n=82) | Durrmeyer | 2018 | JAMA | Double-blind RCT | Multicentric  Preterm and term  Sufentanil+atrac: 29 (26-32)^a^ wks  Propofol:  30 (28-34)^a^ wks | + | 95% | Premature stop of inclusions | 45/87 (51.7) | 75/81 (92.6) | **P<0.001** | 0.6*  (0.5 – 0.7) | -40.9 %*  (-53 to -29) | High | High |
| * Test performed by the working group  ^a^Median (IQR)  ^b^Mean (SD)  Abbreviations: CI, confidence interval; NS, not significant; RCT, randomized controlled trial; wks, weeks of gestation; SpO_2_, oxygen saturation; HR, heart rate; BP, blood pressure; CRT, capillary refill time; GA, gestational age; MBP, mean blood pressure; NIPS, neonatal infant pain scale. | | | | | | | | | | | | | | | | |

| **Supplementary Table 6: Randomized trial comparing premedication with nasal midazolam versus nasal ketamine for tracheal intubation in neonates** | | | | | | | | | | | | | | | | | |  |
| --- | --- | --- | --- | --- | --- | --- | --- | --- | --- | --- | --- | --- | --- | --- | --- | --- | --- | --- |
| **General** | | | | | **Quality** | | | | | | **Patient** | | **Effect** | | | **Synthesis** | |  |
| **Outcome** | **Intervention** | **Author** | **Year** | **Journal** | **Type** | **Consistency (I²)** | | **Direct effect** | **Precision of CI** | **Other**  **bias?** | **Studied group** | **Control group** | **P-value** | **Relative risk** | **Absolute difference** | **Quality** | **Importance** |  |
| **Number of attempts for intubation** | | | | | | | | | | | | | | | | | | |
| Mean (SD) | Midazolam IN (n=27)  vs ketamine IN (n=33) | Milesi | 2017 | Arch Dis Child Fetal Neonatal Ed | Double-blind RCT | Multicentric  Preterm neonates nMDZ:  27.6 (24-34)^a^ wks  nKTM:  28.3 (24-36)^a^  wks  Operators with > 50 intubations | + | | - | Change of medication if failure after 2 doses of the 1st treatment allocated  Interruption of study after planned interim analysis | 1.3 (0.6) | 1.2 (0.5) | P=0.75 | - | +0.1 (-18 to 0.38)* | High | High |  |
| **Duration of intubation** | | | | | | | | | | | | | | | | | | |
| Mean (SD) | Midazolam IN (n=27)  vs ketamine IN (n=33) | Milesi | 2017 | Arch Dis Child Fetal Neonatal Ed | Double-blind RCT | Multicentric  Preterm neonates nMDZ:  27.6 (24-34)^a^ wks  nKTM:  28.3 (24-36)^a^  wks  Operators with > 50 intubations | + | | - | Change of medication if failure after 2 doses of the 1st treatment allocated  Interruption of study after planned interim analysis | 46 (24) s | 43 (19) s | P=0.53 | - | +3 (-8 to 14) s* | High | High |  |
| **Hypoxia** | | | | | | | | | | | | | | | | | | |
| Nadir SpO_2_, Mean (SD) | Midazolam IN (n=27)  vs ketamine IN (n=33) | Milesi | 2017 | Arch Dis Child Fetal Neonatal Ed | Double-blind RCT | Multicentric  Preterm neonates  nMDZ:  27.6 (24-34)^a^ wks  nKTM:  28.3 (24-36)^a^  wks  Operators with > 50 intubations | + | | 95% | Change of medication if failure after 2 doses of the 1st treatment allocated  Interruption of study after planned interim analysis | 72 (14) | 77 (14) | P=0.20 | - | -5 (-12 to 2.3) %* | High | High |  |
| **Bradycardia** | | | | | | | | | | | | | | | | | | |
| HR<100/min,  n/N (%) | Midazolam IN (n=27)  vs ketamine IN (n=33) | Milesi | 2017 | Arch Dis Child Fetal Neonatal Ed | Double-blind RCT | Multicentric  Preterm neonates nMDZ:  27.6 (24-34)^a^ wks  nKTM:  28.3 (24-36)^a^  wks  Operators with > 50 intubations | + | | _ | Change of medication if failure after 2 doses of the 1st treatment allocated  Interruption of study after planned interim analysis | 0/27 (0) | 0/33 (0) | - | - | - | High | High |  |
| **Hypotension** | | | | | | | | | | | | | | | | | | |
| Nadir MBP, Mean (SD) | Midazolam IN (n=27)  vs ketamine IN (n=33) | Milesi | 2017 | Arch Dis Child Fetal Neonatal Ed | Double-blind RCT | Multicentric  Preterm neonates nMDZ:  27.6 (24-34)^a^ wks  nKTM:  28.3 (24-36)^a^  wks  Operators with > 50 intubations | + | | - | Change of medication if failure after 2 doses of the 1st treatment allocated  Interruption of study after planned interim analysis | 26 (9) mm Hg | 28 (8) mm Hg | P=0.65 | - | -2 (-6.4 to 2.4) mmHg* | High | High |  |
| **Pain and comfort** | | | | | | | | | | | | | | | | | | |
| Adequate sedation before intubation (TRACHEA score≤1)  , n/N (%) | Midazolam IN (n=27) vs ketamine IN (n=33) | Milesi | 2017 | Arch Dis Child Fetal Neonatal Ed | Double-blind RCT | Multicentric  Preterm neonates nMDZ:  27.6 (24-34)^a^ wks  nKTM:  28.3 (24-36)^a^  wks  Operators with > 50 intubations | + | | 95% | Change of medication if failure after 2 doses of the 1st treatment allocated  Interruption of study after planned interim analysis | 25/27 (93) | 21/33 (64) | **P=0.004** | 1.5  (1.1 – 1.9)* | +29 (10 to 48) %* | High | High |  |
| Adequate comfort during intubation (FANS<4), n/N (%) | Midazolam IN (n=27) vs ketamine IN (n=33) | Milesi | 2017 | Arch Dis Child Fetal Neonatal Ed | Double-blind RCT | Multicentric  Preterm neonates nMDZ:  27.6 (24-34)^a^ wks  nKTM:  28.3 (24-36)^a^  wks  Operators with > 50 intubations | + | | - | Change of medication if failure after 2 doses of the 1st treatment allocated  Interruption of study after planned interim analysis | 26/27 (96) | 31/33 (95) | P=1.00 | 1.0 (0.9 – 1.1)* | +2.3 (-8 to 13) %* | High | High |  |
| **Intubation conditions** | | | | | | | | | | | | | | | | | | |
| Adequate sedation before intubation (TRACHEA score≤1)  And adequate comfort  during intubation (FANS<4), n/N (%) | Midazolam IN (n=27) vs ketamine IN (n=33) | Milesi | 2017 | Arch Dis Child Fetal Neonatal Ed | Double-blind RCT | Multicentric  Preterm neonates nMDZ:  27.6 (24-34)^a^ wks  nKTM:  28.3 (24-36)^a^  wks  Operators with > 50 intubations | + | | 95% | Change of medication if failure after 2 doses of the 1st treatment allocated  Interruption of study after planned interim analysis | 24/27 (88.9) | 19/33 (57.6) | **P=0.007** | 1.5 (1.1 - 2.1) | +31 (11 to 52) %* | High | Moderate |  |
| * Test performed by the working group  ^a^Mean (range)  Abbreviations: IN, intra nasal; CI, confidence interval; NS, not significant; RCT, randomized controlled trial; wks, weeks of gestation; SpO_2_, oxygen saturation; HR, heart rate; BP, blood pressure; MBP, mean blood pressure; TRACHEA, Tonus, Reactivity, Awareness and Conditions of intubation to Help in Endotracheal intubation Assessment; FANS, faceless acute neonatal pain score | | | | | | | | | | | | | | | | | |  |

| **Supplementary Table 7: Randomized trials comparing premedication versus awake procedure for less invasive surfactant administration (LISA) in neonates** | | | | | | | | | | | | | | | | |
| --- | --- | --- | --- | --- | --- | --- | --- | --- | --- | --- | --- | --- | --- | --- | --- | --- |
| **General** | | | | | **Quality** | | | | | **Patient** | | **Effect** | | | **Synthesis** | |
| **Outcome** | **Intervention** | **Author** | **Year** | **Journal** | **Type** | **Consistency (I²)** | **Direct effect** | **Precision of CI** | **Other**  **bias?** | **Studied group** | **Control group** | **P-value** | **Relative risk** | **Absolute difference** | **Quality** | **Importance** |
| **Failure of LISA procedure** | | | | | | | | | | | | | | | | |
| Intubation within 24h, n/N (%) | Propofol (n=42)  vs no medication (n=36) | Dekker | 2018 | Arch Dis Child Fetal Neonatal Ed | Open RCT | Monocentric  Preterm 26 - 37 wks  Propofol:  29 (27-32)^a^ wks  Control:  29 (28-31)^a^ wks | + | 95% | No | 10/42 (24) | 6/36 (17) | P=0.58 | 1.4*  (0.6 – 3.5) | +7 %*  (-11 to 25) | Moderate | High |
| Intubation within 72h, n/N (%) | Fentanyl (n=17)  vs no medication (n=17) | Sk | 2022 | J Trop Pediatr | Open RCT  Blind analysis | Monocentric  Preterm  Fentanyl:  30.8 (2.1)^b^ wks  Control:  31.6 (2.2)^b^ wks | + | 95% | No | 2/17 (11.8) | 1 /17 (5.9) | P=1.00 | 2.0  (0.2–20.0) | +5.9%*  (-13 to 25) | Moderate | High |
| **Number of laryngoscopies** | | | | | | | | | | | | | | | | |
| N median [IQR] | Propofol (n=42)  vs no medication (n=36) | Dekker | 2018 | Arch Dis Child Fetal Neonatal Ed | Open RCT | Monocentric  Preterm 26 - 37 wks  Propofol:  29 (27-32)^a^ wks  Control:  29 (28-31)^a^ wks | + | - | No | 1 (1-2) | 1 (1-2) | P=0,98 | - | - | Moderate | High |
| >1 laryngoscopy attempt, n/N (%) | Fentanyl (n=17)  vs no medication (n=17) | Sk | 2022 | J Trop Pediatr | Open RCT  Blind analysis | Monocentric  Preterm  Fentanyl:  30.8 (2.1)^b^ wks  Control:  31.6 (2.2)^b^ wks | + | 95% | No | 1/17 (5.9) | 5/17 (29.4) | P= 0.17 | 0.2  (0.03–1.5) | -24%*  (-48 to 0.8) | Moderate | High |
| **Duration of LISA procedure** | | | | | | | | | | | | | | | | |
| Mean  (+/- SD) | Propofol (n=42)  vs no medication (n=36) | Dekker | 2018 | Arch Dis Child Fetal Neonatal Ed | Open RCT | Monocentric  Preterm 26 - 37 wks  Propofol:  29 (27-32)^a^ wks  Control:  29 (28-31)^a^ wks | + | 95% | No | 246.1 (174.8) s | 246.1 (178.4) s | P=0.64 | - | 0 s*  (-80 to 80) | Moderate | High |
| **Hypoxia** | | | | | | | | | | | | | | | | |
| SpO_2_<85%,  n/N (%) | Propofol (n=42)  vs no medication (n=36) | Dekker | 2018 | Arch Dis Child Fetal Neonatal Ed | Open RCT | Monocentric  Preterm 26 - 37 wks  Propofol:  29 (27-32)^a^ wks  Control:  29 (28-31)^a^ wks | + | 95% | No | 38/42 (91) | 25/36 (69) | **P=0.023** | 1.3*  (1.03-1.6) | +21 %*  (3.6 to 39) | Moderate | High |
| SpO2 < 80% >60 s, n/N (%) | Fentanyl (n=17)  vs no medication (n=17) | Sk | 2022 | J Trop Pediatr | Open RCT  Blind analysis | Monocentric  Preterm  Fentanyl:  30.8 (2.1)^b^ wks  Control:  31.6 (2.2)^b^ wks | + | 95% | No | 7/17 (41.2) | 5/17 (29.4) | P=0.72 | 1.4  (0.6–3.5) | +12 %*  (-20 to 44) | Moderate | High |
| **Bradycardia** | | | | | | | | | | | | | | | | |
| HR<100/min, n/N (%) | Propofol (n=42)  vs no medication (n=36) | Dekker | 2018 | Arch Dis Child Fetal Neonatal Ed | Open RCT | Monocentric  Preterm 26 - 37 wks  Propofol:  29 (27-32)^a^ wks  Control:  29 (28-31)^a^ wks | + | 95% | No | 9/42 (21) | 5/35 (14) | P=0,56 | 1.5*  (0.6 – 4.2) | +7.5 %*  (-9 to 24) | Moderate | High |
| HR<100/min, n/N (%) | Fentanyl (n=17)  vs no medication (n=17) | Sk | 2022 | J Trop Pediatr | Open RCT  Blind analysis | Monocentric  Preterm  Fentanyl:  30.8 (2.1)^b^ wks  Control:  31.6 (2.2)^b^ wks | + | 95% | No | 2/17 (11.8) | 2/17 (11.8) | P=1.00 | 1.0  (0.16–6.3) | 0 %*  (-22 to 22) | Moderate | High |
| **Hypotension** | | | | | | | | | | | | | | | | |
| MBP<GA, n/N (%) | Propofol (n=42)  vs no medication (n=36) | Dekker | 2018 | Arch Dis Child Fetal Neonatal Ed | Open RCT | Monocentric  Preterm 26 - 37 wks  Propofol:  29 (27-32)^a^ wks  Control:  29 (28-31)^a^ wks | + | 95% | No | 9/30 (30%) | 2/17 (12%) | P=0.28 | 2.5*  (0.6-10.5) | +18%*  (-4 to 41) | Moderate | High |
| MBP during LISA, mean (+/-SD) | Propofol (n=42)  vs no medication (n=36) | Dekker | 2018 | Arch Dis Child Fetal Neonatal Ed | Open RCT | Monocentric  Preterm 26 - 37 wks  Propofol:  29 (27-32)^a^ wks  Control:  29 (28-31)^a^ wks | + | 95% | No | 35 (7) | 39 (5) | P=0.15 | - | -4 mm Hg*  (-6.8 to -1.2) | Moderate | High |
| MBP 1 min after surfactant, mean (+/-SD) | Fentanyl (n=17)  vs no medication (n=17) | Sk | 2022 | J Trop Pediatr | Open RCT  Blind analysis | Monocentric  Preterm  Fentanyl:  30.8 (2.1)^b^ wks  Control:  31.6 (2.2)^b^ wks | + | 95% | No | 36.53 (6.16) | 37.41 (7.00) | P=0.70 | - | -0.9 mm Hg*  (-5.5 to 3.7) | Moderate | High |
| MBP 5 min after surfactant, mean (+/-SD) | Fentanyl (n=17)  vs no medication (n=17) | Sk | 2022 | J Trop Pediatr | Open RCT  Blind analysis | Monocentric  Preterm  Fentanyl:  30.8 (2.1)^b^ wks  Control:  31.6 (2.2)^b^ wks | + | 95% | No | 35.06 (3.75) | 34.47 (5.51) | P=0.72 | - | +0.6 mm Hg*  (-2.7 to 3.9) | Moderate | High |
| MBP 5 min after surfactant, mean (+/-SD) | Fentanyl (n=17)  vs no medication (n=17) | Sk | 2022 | J Trop Pediatr | Open RCT  Blind analysis | Monocentric  Preterm  Fentanyl:  30.8 (2.1)^b^ wks  Control:  31.6 (2.2)^b^ wks | + | 95% | No | 35.82 (4.72) | 34.29 (3.68) | P=0.30 | - | +1.5 mm Hg* (-1.4 to 4.5) | Moderate | High |
| **Pain and comfort** | | | | | | | | | | | | | | | | |
| Rate of COMFORTneoscore<14, n/N (%) | Propofol (n=42)  vs no medication (n=36) | Dekker | 2018 | Arch Dis Child Fetal Neonatal Ed | Open RCT | Monocentric  Preterm 26 - 37 wks  Propofol:  29 (27-32)^a^ wks  Control:  29 (28-31)^a^ wks | + | 95% | No | 32/42 (76) | 8/36 (22) | **P<0.001** | 3.4*  (1.8 – 6.5) | +54 %*  (35 to 73) | Moderate | High |
| COMFORTneo score, mean (+/-SD) | Propofol (n=42)  vs no medication (n=36) | Dekker | 2018 | Arch Dis Child Fetal Neonatal Ed | Open RCT | Monocentric  Preterm 26 - 37 wks  Propofol:  29 (27-32)^a^ wks  Control:  29 (28-31)^a^ wks | + | 95% | No | 12 (3) | 17 (4) | **P<0.001** | - | -5*  (-6.6 to -3.4) | Moderate | High |
| Rate of R-PIPP score<12, n/N (%) | Fentanyl (n=17)  vs no medication (n=17) | Sk | 2022 | J Trop Pediatr | Open RCT  Blind analysis | Monocentric  Preterm  Fentanyl:  30.8 (2.1)^b^ wks  Control:  31.6 (2.2)^b^ wks | + | 95% | No | 15/17 (88.2) | 8/17 (47.1) | **P=0.025** | 1.9*  (1.1 – 3.2) | +41%*  (13 to 69) | Moderate | High |
| R-PIPP score, median ([IQR] | Fentanyl (n=17)  vs no medication (n=17) | Sk | 2022 | J Trop Pediatr | Open RCT  Blind analysis | Monocentric  Preterm  Fentanyl:  30.8 (2.1)^b^ wks  Control:  31.6 (2.2)^b^ wks | + | - | No | 9 [7–10] | 12 [9–13] | **P=0.004** | - | - | Moderate | High |
| **Conditions of LISA procedure** | | | | | | | | | | | | | | | | |
| Intubation during LISA, n/N (%) | Propofol (n=42)  vs no medication (n=36) | Dekker | 2018 | Arch Dis Child Fetal Neonatal Ed | Open RCT | Monocentric  Preterm 26 - 37 wks  Propofol:  29 (27-32)^a^ wks  Control:  29 (28-31)^a^ wks | + | 95% | No | 1/42 (2) | 4/36 (11) | P=0.18 | 0.2*  (0.03-1.8) | -8.7%*  (-20 to 2.5) | Moderate | High |
| Intubation during LISA, n/N (%) | Fentanyl (n=17)  vs no medication (n=17) | Sk | 2022 | J Trop Pediatr | Open RCT  Blind analysis | Monocentric  Preterm  Fentanyl:  30.8 (2.1)^b^ wks  Control:  31.6 (2.2)^b^ wks | + | 95% | No | 1/17 (5.9) | 0/17 (0) | P=1.00 | 3.0  (0.13–68.8) | +5.9%*  (-5.3 to 17) | Moderate | High |
| **In-hospital morbidity and mortality** | | | | | | | | | | | | | | | | |
| Pneumothorax, n/N (%) | Propofol (n=42)  vs no medication (n=36) | Dekker | 2018 | Arch Dis Child Fetal Neonatal Ed | Open RCT | Monocentric  Preterm 26 - 37 wks  Propofol:  29 (27-32)^a^ wks  Control:  29 (28-31)^a^ wks | + | 95% | No | 3/42 (7) | 1/36 (3) | P=0.62 | 2.6*  (0.3 – 24) | +4.4%*  (-5.1 to 14) | Moderate | Moderate |
| Pulmonary hemorrhage, n/N (%) | Propofol (n=42)  vs no medication (n=36) | Dekker | 2018 | Arch Dis Child Fetal Neonatal Ed | Open RCT | Monocentric  Preterm 26 - 37 wks  Propofol:  29 (27-32)^a^ wks  Control:  29 (28-31)^a^ wks | + | 95% | No | 1/42 (2) | 0/36 (0) | P=1.00 | - | +2.4%*  (-2.2 to 7.0) | Moderate | Moderate |
| Intra-ventricular hemorrhage grade ≥3,  n/N (%) | Propofol (n=42)  vs no medication (n=36) | Dekker | 2018 | Arch Dis Child Fetal Neonatal Ed | Open RCT | Monocentric  Preterm 26 - 37 wks  Propofol:  29 (27-32)^a^ wks  Control:  29 (28-31)^a^ wks | + | 95% | No | 2/42 (5) | 0/36 (0) | P=0.50 | - | +4.8%*  (-1.7 to 11) | Moderate | Moderate |
| Death, n/N (%) | Propofol (n=42)  vs no medication (n=36) | Dekker | 2018 | Arch Dis Child Fetal Neonatal Ed | Open RCT | Monocentric  Preterm 26 - 37 wks  Propofol:  29 (27-32)^a^ wks  Control:  29 (28-31)^a^ wks | + | 95% | No | 1/42 (2) | 1/36 (3) | P=1.00 | 0.9*  (0.06 -13) | -0.4%*  (-7.5 to 6.7) | Moderate | Low |
| Intra-ventricular hemorrhage grade ≥3,  n/N (%) | Fentanyl (n=17)  vs no medication (n=17) | Sk | 2022 | J Trop Pediatr | Open RCT  Blind analysis | Monocentric  Preterm  Fentanyl:  30.8 (2.1)^b^ wks  Control:  31.6 (2.2)^b^ wks | + | 95% | No | 1/17 (5.9) | 1/17 (5.9) | P=1.00 | 1.0  (0.07–14.7) | 0%*  (-16 to 16) | Moderate | Moderate |
| BPD or death, n/N (%) | Fentanyl (n=17)  vs no medication (n=17) | Sk | 2022 | J Trop Pediatr | Open RCT  Blind analysis | Monocentric  Preterm  Fentanyl:  30.8 (2.1)^b^ wks  Control:  31.6 (2.2)^b^ wks | + | 95% | No | 6/17 (35.3) | 4/17 (23.5) | P=0.71 | 1.5  (0.5–4.4) | +12%*  (-19 to 42) | Moderate | Low |
| * Test performed by the working group  ^a^Median (IQR)  ^b^ Mean (SD)  Abbreviations: LISA, less invasive surfactant administration; CI, confidence interval; wks, weeks of gestation; NS, not significant; GA, gestational age; RCT, randomized controlled trial; SpO_2_, oxygen saturation; HR, heart rate; BP, blood pressure; MBP, mean blood pressure; R-PIPP, revised premature infant pain profile; BPD, broncho-pulmonary dysplasia. | | | | | | | | | | | | | | | | |

| **Supplementary Table 8:** **Randomized trial comparing premedication with opioids versus awake procedure for less invasive surfactant administration (LISA) in neonates** | | | | | | | | | | | | | | | | |
| --- | --- | --- | --- | --- | --- | --- | --- | --- | --- | --- | --- | --- | --- | --- | --- | --- |
| **General** | | | | | **Quality** | | | | | **Patient** | | **Effect** | | | **Synthesis** | |
| **Outcome** | **Intervention** | **Author** | **Year** | **Journal** | **Type** | **Consistency (I²)** | **Direct effect** | **Precision of CI** | **Other**  **bias?** | **Studied group** | **Control group** | **P-value** | **Relative risk** | **Absolute difference** | **Quality** | **Importance** |
| **Failure of LISA procedure** | | | | | | | | | | | | | | | | |
| Intubation within 72h, n/N (%) | Fentanyl (n=17)  vs no medication (n=17) | Sk | 2022 | J Trop Pediatr | Open RCT  Blind analysis | Monocentric  Preterm  Fentanyl:  30.8 (2.1)^a^ wks  Control:  31.6 (2.2)^a^ wks | + | 95% | No | 2/17 (11.8) | 1 /17 (5.9) | P=1.00 | 2.0  (0.2–20.0) | +5.9%*  (-13 to 25) | Moderate | High |
| **Number of laryngoscopies** | | | | | | | | | | | | | | | | |
| >1 laryngoscopy attempt, n/N (%) | Fentanyl (n=17)  vs no medication (n=17) | Sk | 2022 | J Trop Pediatr | Open RCT  Blind analysis | Monocentric  Preterm  Fentanyl:  30.8 (2.1)^a^ wks  Control:  31.6 (2.2)^a^ wks | + | 95% | No | 1/17 (5.9) | 5/17 (29.4) | P= 0.17 | 0.2  (0.03–1.5) | -24%*  (-48 to 0.8) | Moderate | High |
| **Duration of LISA procedure: not evaluated** | | | | | | | | | | | | | | | | |
| **Hypoxia** | | | | | | | | | | | | | | | | |
| SpO2 < 80% >60 s, n/N (%) | Fentanyl (n=17)  vs no medication (n=17) | Sk | 2022 | J Trop Pediatr | Open RCT  Blind analysis | Monocentric  Preterm  Fentanyl:  30.8 (2.1)^a^ wks  Control:  31.6 (2.2)^a^ wks | + | 95% | No | 7/17 (41.2) | 5/17 (29.4) | P=0.72 | 1.4  (0.6–3.5) | +12 %*  (-20 to 44) | Moderate | High |
| **Bradycardia** | | | | | | | | | | | | | | | | |
| HR<100/min, n/N (%) | Fentanyl (n=17)  vs no medication (n=17) | Sk | 2022 | J Trop Pediatr | Open RCT  Blind analysis | Monocentric  Preterm  Fentanyl:  30.8 (2.1)^a^ wks  Control:  31.6 (2.2)^a^ wks | + | 95% | No | 2/17 (11.8) | 2/17 (11.8) | P=1.00 | 1.0  (0.16–6.3) | 0 %*  (-22 to 22) | Moderate | High |
| **Hypotension** | | | | | | | | | | | | | | | | |
| MBP 1 min after surfactant, Mean (+/-SD) | Fentanyl (n=17)  vs no medication (n=17) | Sk | 2022 | J Trop Pediatr | Open RCT  Blind analysis | Monocentric  Preterm  Fentanyl:  30.8 (2.1)^a^ wks  Control:  31.6 (2.2)^a^ wks | + | 95% | No | 36.53 (6.16) | 37.41 (7.00) | P=0.70 | - | -0.9 mm Hg*  (-5.5 to 3.7) | Moderate | High |
| MBP 5 min after surfactant, Mean (+/-SD) | Fentanyl (n=17)  vs no medication (n=17) | Sk | 2022 | J Trop Pediatr | Open RCT  Blind analysis | Monocentric  Preterm  Fentanyl:  30.8 (2.1)^a^ wks  Control:  31.6 (2.2)^a^ wks | + | 95% | No | 35.06 (3.75) | 34.47 (5.51) | P=0.72 | - | +0.6 mm Hg*  (-2.7 to 3.9) | Moderate | High |
| MBP 5 min after surfactant, Mean (+/-SD) | Fentanyl (n=17)  vs no medication (n=17) | Sk | 2022 | J Trop Pediatr | Open RCT  Blind analysis | Monocentric  Preterm  Fentanyl:  30.8 (2.1)^a^ wks  Control:  31.6 (2.2)^a^ wks | + | 95% | No | 35.82 (4.72) | 34.29 (3.68) | P=0.30 | - | +1.5 mm Hg* (-1.4 to 4.5) | Moderate | High |
| **Pain and comfort** | | | | | | | | | | | | | | | | |
| Rate of R-PIPP score<12, n/N (%) | Fentanyl (n=17)  vs no medication (n=17) | Sk | 2022 | J Trop Pediatr | Open RCT  Blind analysis | Monocentric  Preterm  Fentanyl:  30.8 (2.1)^a^ wks  Control:  31.6 (2.2)^a^ wks | + | 95% | No | 15/17 (88.2) | 8/17 (47.1) | **P=0.025** | 1.9*  (1.1 – 3.2) | +41%*  (13 to 69) | Moderate | High |
| R-PIPP score, Median ([IQR] | Fentanyl (n=17)  vs no medication (n=17) | Sk | 2022 | J Trop Pediatr | Open RCT  Blind analysis | Monocentric  Preterm  Fentanyl:  30.8 (2.1)^a^ wks  Control:  31.6 (2.2)^a^ wks | + | - | No | 9 [7–10] | 12 [9–13] | **P=0.004** | - | - | Moderate | High |
| **Conditions of LISA procedure** | | | | | | | | | | | | | | | | |
| Intubation during LISA, n/N (%) | Fentanyl (n=17)  vs no medication (n=17) | Sk | 2022 | J Trop Pediatr | Open RCT  Blind analysis | Monocentric  Preterm  Fentanyl:  30.8 (2.1)^a^ wks  Control:  31.6 (2.2)^a^ wks | + | 95% | No | 1/17 (5.9) | 0/17 (0) | P=1.00 | 3.0  (0.13–68.8) | +5.9%*  (-5.3 to 17) | Moderate | High |
| **In-hospital morbidity and mortality** | | | | | | | | | | | | | | | | |
| Intra-ventricular hemorrhage ≥3,  n/N (%) | Fentanyl (n=17)  vs no medication (n=17) | Sk | 2022 | J Trop Pediatr | Open RCT  Blind analysis | Monocentric  Preterm  Fentanyl:  30.8 (2.1)^a^ wks  Control:  31.6 (2.2)^a^ wks | + | 95% | No | 1/17 (5.9) | 1/17 (5.9) | P=1.00 | 1.0  (0.07–14.7) | 0%*  (-16 to 16) | Moderate | Moderate |
| BPD or death, n/N (%) | Fentanyl (n=17)  vs no medication (n=17) | Sk | 2022 | J Trop Pediatr | Open RCT  Blind analysis | Monocentric  Preterm  Fentanyl:  30.8 (2.1)^a^ wks  Control:  31.6 (2.2)^a^ wks | + | 95% | No | 6/17 (35.3) | 4/17 (23.5) | P=0.71 | 1.5  (0.5–4.4) | +12%*  (-19 to 42) | Moderate | Low |
| * Test performed by the working group  ^a^ Mean (SD)  Abbreviations: LISA, less invasive surfactant administration; CI, confidence interval; wks, weeks of gestation; GA, gestational age; RCT, randomized controlled trial; SpO_2_, oxygen saturation; HR, heart rate; MBP, mean blood pressure; R-PIPP, revised premature infant pain profile; BPD, broncho-pulmonary dysplasia. | | | | | | | | | | | | | | | | |

| **Supplementary Table *9*: Randomized trials comparing premedication with propofol versus awake procedure for less invasive surfactant administration (LISA) in neonates** | | | | | | | | | | | | | | | | |
| --- | --- | --- | --- | --- | --- | --- | --- | --- | --- | --- | --- | --- | --- | --- | --- | --- |
| **General** | | | | | **Quality** | | | | | **Patient** | | **Effect** | | | **Synthesis** | |
| **Outcome** | **Intervention** | **Author** | **Year** | **Journal** | **Type** | **Consistency (I²)** | **Direct effect** | **Precision of CI** | **Other**  **bias?** | **Studied group** | **Control group** | **P-value** | **Relative risk** | **Absolute difference** | **Quality** | **Importance** |
| **Failure of LISA procedure** | | | | | | | | | | | | | | | | |
| Intubation within 24h, n/N (%) | Propofol (n=42)  vs no medication (n=36) | Dekker | 2018 | Arch Dis Child Fetal Neonatal Ed | Open RCT | Monocentric  Preterm 26 - 37 wks  Propofol:  29 (27-32)^a^ wks  Control:  29 (28-31)^a^ wks | + | 95% | No | 10/42 (24) | 6/36 (17) | P=0.58 | 1.4*  (0.6 – 3.5) | +7 %*  (-11 to 25) | Moderate | High |
| **Number of laryngoscopies** | | | | | | | | | | | | | | | | |
| N median [IQR] | Propofol (n=42)  vs no medication (n=36) | Dekker | 2018 | Arch Dis Child Fetal Neonatal Ed | Open RCT | Monocentric  Preterm 26 - 37 wks  Propofol:  29 (27-32)^a^ wks  Control:  29 (28-31)^a^ wks | + | - | No | 1 (1-2) | 1 (1-2) | P=0,98 | - | - | Moderate | High |
| **Duration of LISA procedure** | | | | | | | | | | | | | | | | |
| Mean  (+/- SD) | Propofol (n=42)  vs no medication (n=36) | Dekker | 2018 | Arch Dis Child Fetal Neonatal Ed | Open RCT | Monocentric  Preterm 26 - 37 wks  Propofol:  29 (27-32)^a^ wks  Control:  29 (28-31)^a^ wks | + | 95% | No | 246.1 (174.8) s | 246.1 (178.4) s | P=0.64 | - | 0 s*  (-80 to 80) | Moderate | High |
| **Hypoxia** | | | | | | | | | | | | | | | | |
| SpO_2_<85%,  n/N (%) | Propofol (n=42)  vs no medication (n=36) | Dekker | 2018 | Arch Dis Child Fetal Neonatal Ed | Open RCT | Monocentric  Preterm 26 - 37 wks  Propofol:  29 (27-32)^a^ wks  Control:  29 (28-31)^a^ wks | + | 95% | No | 38/42 (91) | 25/36 (69) | **P=0.023** | 1.3*  (1.03-1.6) | +21 %*  (3.6 to 39) | Moderate | High |
| **Bradycardia** | | | | | | | | | | | | | | | | |
| HR<100/min, n/N (%) | Propofol (n=42)  vs no medication (n=36) | Dekker | 2018 | Arch Dis Child Fetal Neonatal Ed | Open RCT | Monocentric  Preterm 26 - 37 wks  Propofol:  29 (27-32)^a^ wks  Control:  29 (28-31)^a^ wks | + | 95% | No | 9/42 (21) | 5/35 (14) | P=0,56 | 1.5*  (0.6 – 4.2) | +7.5 %*  (-9 to 24) | Moderate | High |
| **Hypotension** | | | | | | | | | | | | | | | | |
| MBP<GA, n/N (%) | Propofol (n=42)  vs no medication (n=36) | Dekker | 2018 | Arch Dis Child Fetal Neonatal Ed | Open RCT | Monocentric  Preterm 26 - 37 wks  Propofol:  29 (27-32)^a^ wks  Control:  29 (28-31)^a^ wks | + | 95% | No | 9/30 (30%) | 2/17 (12%) | P=0.28 | 2.5*  (0.6-10.5) | +18%*  (-4 to 41) | Moderate | High |
| MBP during LISA, mean (+/-SD) | Propofol (n=42)  vs no medication (n=36) | Dekker | 2018 | Arch Dis Child Fetal Neonatal Ed | Open RCT | Monocentric  Preterm 26 - 37 wks  Propofol:  29 (27-32)^a^ wks  Control:  29 (28-31)^a^ wks | + | 95% | No | 35 (7) | 39 (5) | P=0.15 | - | -4 mm Hg*  (-6.8 to -1.2) | Moderate | High |
| **Pain and comfort** | | | | | | | | | | | | | | | | |
| Rate of COMFORTneoscore<14, n/N (%) | Propofol (n=42)  vs no medication (n=36) | Dekker | 2018 | Arch Dis Child Fetal Neonatal Ed | Open RCT | Monocentric  Preterm 26 - 37 wks  Propofol:  29 (27-32)^a^ wks  Control:  29 (28-31)^a^ wks | + | 95% | No | 32/42 (76) | 8/36 (22) | **P<0.001** | 3.4*  (1.8 – 6.5) | +54 %*  (35 to 73) | Moderate | High |
| COMFORTneo score, mean (+/-SD) | Propofol (n=42)  vs no medication (n=36) | Dekker | 2018 | Arch Dis Child Fetal Neonatal Ed | Open RCT | Monocentric  Preterm 26 - 37 wks  Propofol:  29 (27-32)^a^ wks  Control:  29 (28-31)^a^ wks | + | 95% | No | 12 (3) | 17 (4) | **P<0.001** | - | -5*  (-6.6 to -3.4) | Moderate | High |
| **Conditions of LISA procedure** | | | | | | | | | | | | | | | | |
| Intubation during LISA, n/N (%) | Propofol (n=42)  vs no medication (n=36) | Dekker | 2018 | Arch Dis Child Fetal Neonatal Ed | Open RCT | Monocentric  Preterm 26 - 37 wks  Propofol:  29 (27-32)^a^ wks  Control:  29 (28-31)^a^ wks | + | 95% | No | 1/42 (2) | 4/36 (11) | P=0.18 | 0.2*  (0.03-1.8) | -8.7%*  (-20 to 2.5) | Moderate | High |
| **In-hospital morbidity and mortality** | | | | | | | | | | | | | | | | |
| Pneumothorax, n/N (%) | Propofol (n=42)  vs no medication (n=36) | Dekker | 2018 | Arch Dis Child Fetal Neonatal Ed | Open RCT | Monocentric  Preterm 26 - 37 wks  Propofol:  29 (27-32)^a^ wks  Control:  29 (28-31)^a^ wks | + | 95% | No | 3/42 (7) | 1/36 (3) | P=0.62 | 2.6*  (0.3 – 24) | +4.4%*  (-5.1 to 14) | Moderate | Moderate |
| Pulmonary hemorrhage, n/N (%) | Propofol (n=42)  vs no medication (n=36) | Dekker | 2018 | Arch Dis Child Fetal Neonatal Ed | Open RCT | Monocentric  Preterm 26 - 37 wks  Propofol:  29 (27-32)^a^ wks  Control:  29 (28-31)^a^ wks | + | 95% | No | 1/42 (2) | 0/36 (0) | P=1.00 | - | +2.4%*  (-2.2 to 7.0) | Moderate | Moderate |
| Intra-ventricular hemorrhage grade ≥3,  n/N (%) | Propofol (n=42)  vs no medication (n=36) | Dekker | 2018 | Arch Dis Child Fetal Neonatal Ed | Open RCT | Monocentric  Preterm 26 - 37 wks  Propofol:  29 (27-32)^a^ wks  Control:  29 (28-31)^a^ wks | + | 95% | No | 2/42 (5) | 0/36 (0) | P=0.50 | - | +4.8%*  (-1.7 to 11) | Moderate | Moderate |
| Death, n/N (%) | Propofol (n=42)  vs no medication (n=36) | Dekker | 2018 | Arch Dis Child Fetal Neonatal Ed | Open RCT | Monocentric  Preterm 26 - 37 wks  Propofol:  29 (27-32)^a^ wks  Control:  29 (28-31)^a^ wks | + | 95% | No | 1/42 (2) | 1/36 (3) | P=1.00 | 0.9*  (0.06 -13) | -0.4%*  (-7.5 to 6.7) | Moderate | Low |
| * Test performed by the working group  ^a^Median (IQR)  Abbreviations: LISA, less invasive surfactant administration; CI, confidence interval; GA, gestational age; RCT, randomized controlled trial; SpO_2_, oxygen saturation; HR, heart rate; MBP, mean blood pressure. | | | | | | | | | | | | | | | | |
